# Work environment of nurses in primary health care in Manaus: an analytical cross-sectional study*

**DOI:** 10.1590/1980-220X-REEUSP-2025-0280en

**Published:** 2026-03-06

**Authors:** Lucas Lorran Costa de Andrade, Laura Cavalcanti de Farias Brehmer, Elisiane Lorenzini, Sabrina Blasius Faust, Wagner Ferreira Monteiro, Letícia de Lima Trindade, Ronilson Ferreira Freitas, Flávia Regina Souza Ramos

**Affiliations:** 1 Universidade Federal de Santa Catarina, Departamento de Enfermagem, Florianópolis, SC, Brazil.; 2 Universidade Federal do Amazonas, Departamento de Saúde Coletiva, Manaus, AM, Brazil.; 3 Universidade do Estado do Amazonas, Departamento de Enfermagem, Manaus, AM, Brazil.; 4 Universidade do Estado de Santa Catarina, Departamento de Enfermagem, Chapecó, SC, Brazil.

**Keywords:** Primary Health Care, Work, Health Facility Environment, Nurses

## Abstract

**Objective:**

To analyze the dimensions of the work environment of primary health care nurses (PHC) and its relationship with these professionals’ sociodemographic characteristics.

**Method:**

This was a cross-sectional, analytical study conducted between April and October 2024 with 243 PHC nurses in Manaus. A questionnaire and the Workplace Environment Assessment Scale were used. Descriptive and inferential analysis was performed.

**Results:**

The overall assessment of the work environment was average, with a score of 98.1. The average score for the Motivation for Work dimension was 47.2. The Workplace Safety dimension had the lowest average (14.6), classified as “attention”, and Strategic Management for Healthy Work, with an average of 36.3, was classified as having warning points. A negative correlation was found between income and motivation, length of time working in PHC and management, age/length of work and job security. Temporary nurses evaluated strategic management more positively.

**Conclusion:**

Weaknesses were identified in security and strategic management, highlighting the need for improvements in infrastructure, participatory management, and protection of professionals’ health to promote a safer and healthier work environment.

## INTRODUCTION

Primary Health Care (PHC), also known as Basic Care in Brazil, is considered the main entry point to the Brazilian Public Health System (*SUS*) and plays a central role in health promotion, disease prevention, and continuous care for the population. This level of care has become a central axis in health policies, making it essential to analyze the actors, scenarios, and how the complexity of workplace demands can affect the health and well-being of professionals, including nursing ones^(1,2)^.

Nurses in PHC services assume distinct responsibilities, including care coordination, chronic disease management, health education, clinical and administrative activities, as well as conduction of home visits as an integral part of their duties in the care provided at health units^(3)^. Despite the extensive responsibilities and importance of nurses’ role in PHC, the reality of these professionals’ work is marked by challenges that can compromise the quality of care, such as overload, lack of resources and inadequate infrastructure, and workplace violence, which triggers effects on physical and mental health, even generating moral suffering^(4,5)^.

In certain regions, these challenges can be even more pronounced due to the specificities of the local context, such as the large territorial extension, cultural diversity, and social inequalities^(6,7)^. In the Brazilian Amazon region, although there have been advances in the planning and expansion of primary health care, the health professionals working there, especially nurses, still suffer from work overload. Support for overcoming challenging working conditions is a significant obstacle, as is adapting the work environment for healthcare professionals so that they understand the specific characteristics of the region^(8)^.

Given this challenging context, building a healthy work environment (HWE) emerges as a fundamental need for nurses’ physical and emotional well-being. According to the World Health Organization (WHO), a HWE is one in which workers and managers collaborate to promote and protect the health, safety, and well-being of all, as well as ensuring the sustainability of the work environment^(9)^.

Researchers in the field of HWE emphasize the importance of considering both objective dimensions, such as the physical environment and working conditions, and subjective dimensions, which include symbolic and ethical factors^(10)^. In other words, by providing adequate physical conditions, professional recognition, institutional support, development opportunities, and a positive organizational climate, it is possible to promote nurses’ physical, mental, and social health. This measure contributes to improving these professionals’ quality of life and positively impacts the quality of care provided to the population^(11)^.

Based on studies conducted on the evaluation of work environments in PHC, the use of the Workplace Environment Assessment Scale (WES-PHC) presents important axes for understanding the impact of the work environment on workers’ health and well-being. The use of this tool helps in identifying critical areas and in making informed decisions about changes in the work environment, aiming to improve the quality of life of workers and increase the productivity and efficiency of organizations^(10,12)^.

Although the literature points to the challenges faced by nurses in PHC, the vast majority of these studies are limited to the South and Southeast regions of Brazil and the hospital sector^(13,14)^. This overview creates a bias in understanding, widening the gap in relation to regions with unique contexts, such as the North. In this region, local factors, whether logistical, cultural, or resource-related, can have an impact on the work environment. Thus, it is clear that there is a scarcity of research that, starting from the particularities of the Amazon region, seeks to understand nurses’ perceptions and identify the contextual elements shaping their professional daily lives^(15)^.

Given the complexity presented and the need for further knowledge, the objective of this study is to analyze the dimensions of the work environment of primary health care nurses and its relationship with these professionals’ sociodemographic characteristics.

## METHOD

### Design of Study

This is an analytical cross-sectional study, conducted between April and October 2024, reported according to the *Strengthening the Reporting of Observational Studies in Epidemiology* (STROBE) guidelines.

### Population, Local and Selection Criteria

The research was conducted with PHC nurses in the municipality of Manaus, capital of the state of Amazonas, which is administratively organized into five Health Districts (DISA): North, East, West, South and Rural, with a total of 288 Primary Health Care Units (*UBS*) and 500 nursing professionals, according to data from the National Health Facility Census (Accessed on 02/05/2024). The eligible population was considered to be all nursing professionals who were working in *UBS*s during the data collection period, for at least three months. Nurses who exclusively held administrative or management positions outside *UBS*s, as well as those who were absent for any reason during the data collection period, were excluded.

### Sample Definition

Proportional stratified sampling was employed, ensuring that the number of participating nurses adequately represented each health district (North, East, West, South, and Rural). The target population consisted of 500 PHC nurses, distributed as follows: North District with 140 nurses (28%), East District with 121 (24.2%), West District with 106 (21.2%), South District with 108 (21.6%), and Rural District with 25 (5%).

The sample size calculation was performed using the SestatNet^®^ system, employing the following parameters: a sampling error of 5%, a confidence level of 95%, and a margin of error of 10%. Based on these criteria, the sample size was defined as 239 nurses, distributed proportionally among the districts, as follows: 67 nurses from the North District, 58 from the East District, 51 from the West District, 52 from the South District, and 11 from the Rural District. This stratification process ensured that the sample accurately reflected the proportions of the original population.

### Data Collection

Data collection was carried out through the application of a questionnaire developed by the authors, consisting of socio-professional questions, with the following variables: age, sex (assigned at birth), marital status, institution where they work, weekly workload, employment status, time since graduation, length of experience in PHC, other work relationships, role in management, and professional training. Furthermore, the Primary Health Care Workplace Environment Assessment Scale (WES-PHC), created and validated in Brazil, was used. This scale adopted the best practices defined by the American Educational Research Association (AERA) and achieved internal structural validity and reliability^(10,12)^. The scale consists of 36 items, distributed across three dimensions, which reflect the perceptions or feelings of professionals in their work environment: Motivation for work (15 questions); Safety in the work environment (6 questions); and Strategic management for healthy work (15 questions). For each item, the participant indicates how often the situation/perception/feeling occurs on a scale of 0 to 4, where 0 means never and 4 means always.

To assess the work environment of nurses using the WES-PHC, the average scores for the overall score and the three dimensions were considered: motivation for work, safety in the work environment, and strategic management for healthy work. Each dimension has four classification ranges: critical (concerning condition, with a score close to zero), threat (moderate risk, requiring improvements), attention (alert points, but not critical), and healthy (adequate and positive environment). In the dimension of motivation for work and strategic management, scores range from 0 to 60, with scores of 45 or higher considered healthy, 30 to 44 indicating attention, 15 to 29 indicating threat, and below 15 indicating critical. In the workplace safety dimension, scores range from 0 to 24, with healthy scores starting at 18, attention between 12 and 17, threat from 6 to 11, and critical below 6. The overall score ranges from 0 to 144, classified as healthy from 108, concern between 72 and 107, threat from 36 to 71, and critical below 36^(10,12)^.

Participants were initially contacted via the WhatsApp^®^ application, using numbers provided by district supervisors or directors. Through this contact, they received a link to read and accept the Free Informed Consent Form, and the questionnaire was made available via *Google Forms.* In some cases, it was necessary to collect data in person from the participants, scheduling a meeting at the UBS at a time convenient for the professional, so as not to interfere with their activities.

### Data Analysis and Treatment

The software *Statistical Package for Social Science* SPSS version 23 was used to perform the analyses. Initially, the categorical variables were presented in absolute (n) and relative (%) frequencies. Continuous variables were described using means, standard deviations (SD), medians, and interquartile ranges (P25-P75), according to the data distribution.

The normality of the data in each variable was assessed using the Shapiro-Wilk test. To analyze the quality of the work environment according to the three dimensions of the WES-PHC (Motivation for Work, Safety in the Work Environment, and Strategic Management for Healthy Work), the mean and standard deviation were calculated. Pearson’s chi-square test was used to analyze associations between categorical variables (sociodemographic characteristics and perceptions related to the work environment) and Spearman’s correlation analysis to identify possible correlations between continuous variables (age, training time, professional experience) and the WES-PHC dimensions. Analysis of Variance (ANOVA) was also used to compare means between three or more groups, such as marital status, education level, health district, and others. The results were organized into tables, highlighting the significant associations considering p < 0.05.

### Ethical Aspects

The research was conducted in accordance with the Guidelines and Regulatory Standards for Research Involving Human Beings, as per Resolution No. 466/12 of the National Health Council, and approved by the Research Ethics Committee of the Universidade Federal de Santa Catarina in 2024, under opinion number 6.735.782. All participants gave their formal consent by signing the Free Informed Consent Form before participating in the study.

## RESULTS

The study sample consisted of 243 PHC nurses, with a mean age of 43.8 years, who predominantly have specialization-level training (83.5%). Female nurses predominated (87.2%), who were married or in a common-law marriage (55.1%), had a median time of 16 years since graduation, had an employment relationship (86%), and temporary (47.3%) and permanent contract (46.9%) employment relationships prevailed. Predominantly, nurses had a weekly workload of 40 hours (70.8%), and the median length of service at their current institution was four years. Regarding income, the largest percentage of participants (49%) earned between five and six minimum wages, as shown in Table 1.

**Table 1:** Sociodemographic and professional characteristics of primary health care nurses – Manaus, AM, Brazil, 2024.

Variables	n (%)
Age, years – mean ± SD	43.8 ± 9.7
Sex – n (%)	
Male	31 (12.8)
Female	212 (87.2)
Marital Status – n (%)	
Single	81 (33.3)
Married/Common law marriage	134 (55,1)
Divorced	26 (10,7)
Widower	2 (0.8)
Time since graduation (years) – median (P25–P75)	16 (10–20)
Course – n (%)	
Undergraduate course	10 (4.1)
Residence	15 (6.2)
Graduate certificate course	203 (83.5)
Master’s/Doctorate	15 (6.2)
Number of employment relationships – n (%)	
One	209 (86.0)
Two	34 (14.0)
Type of employment relationship – n (%)	
CLT (Consolidation of Labor Laws – a decree which governs labor relations in Brazil)	14 (5.8)
Permanent contract	114 (46.9)
Temporary	115 (47.3)
Income – n (%)	
1 to 2 minimum wages	2 (0.8)
3 to 4 minimum wages	32 (13.2)
5 to 6 minimum wages	119 (49.0)
7 to 8 minimum wages	68 (28.0)
9 or more minimum wages	22 (9.1)
Length of service in Primary Care (years) – median (P25–P75) District – n (%)	6 (3–18)
East	58 (23.9)
West	54 (22.2)
North	67 (27.6)
South	53 (21.8)
Rural	11 (4.5)
Length of service at the current institution (years) – median (P25 – P75)	4 (1–6)
Weekly workload – n (%)	
20 h	3 (1.2)
30 h	62 (25.5)
40 h	172 (70.8)
60 h	6 (2.5)
Healthcare team you work with – n (%)	
Direction	8 (3.3)
PHC team	65 (26.7)
FHS team	170 (70.0)
Holds a management position in the PHC – n (%)	
Yes	55 (22.6)
No	168 (69.1)
Not at the moment, but I have occupied it before	20 (8.2)

^*^SD = standard deviation.

The results showed that the Motivation for Work dimension presented the best indicators, with a total score of 47.2 (±8.86) and an average of 3.14 per item, which reflects a generally positive perception among nurses regarding motivation. In contrast, Workplace Safety was rated as “attention”, with a total score of 14.6 (±5.66) and an average of 2.43 per item, which shows that there are weaknesses in the physical structure and workplace protection. Strategic Management for Healthy Work also showed results with warning points, but not critical ones, with a total score of 36.3 (±12.0) and an average of 2.42 per item, which indicates deficiencies in worker health protection policies.

The combined overall score for the three dimensions was 98.1 (±22.6), with an average of 2.72 per item, placing it in the “requiring attention” range (73–108) according to the WES-PHC parameters. Overall, the assessed environment is close to satisfactory, but it reveals threats and elements that require attention and intervention in two dimensions. Each dimension follows this result (attention) despite the differences identified. Table 2 presents the results of the three dimensions provided by the WES-PHC, with the total scores for each dimension and the nurses’ overall perception of their work environment.

**Table 2:** Results of the assessment of the work environment in primary health care by items and construct – Manaus, AM, Brazil, 2024.

Items	Mean ± SD^*^
**Motivation for work**
What I do/practice in my work gives meaning to my life.	3.55 ± 0.72
I feel engaged and motivated when I’m working.	3.38 ± 0.79
My colleagues who work with me have knowledge and skills consistent with the work they do.	3.14 ± 0.78
In my workplace, the organization of the work process is aligned with the objectives of Primary Health Care (PHC).	3.12 ± 0.86
I have access to the information necessary to carry out my activities.	3.41 ± 0.77
There is adequate and respectful communication between the team and the Unit’s management, promoting participation in decisions and work organization.	3.18 ± 0.96
My colleagues act in an integrated (participatory) way in the work environment.	2.99 ± 0.88
In my workplace, we are able to resolve conflicts in a way that does not compromise work objectives.	3.14 ± 0.80
There is adequate communication between the professionals at my workplace and the users, which promotes the development of the work.	3.06 ± 0.82
There is coordination between the management of the Health Unit or the representative agencies of Primary Care management with associations and other collective organizations of workers (workers’ union, professional councils, health council).	2.59 ± 1.14
When I’m working, I feel comfortable and free from constant observation by management, coordinators, colleagues, or users.	3.15 ± 0.96
I am satisfied with the image that users have of my work.	3.53 ± 0.69
I believe that my colleagues are valued by the health unit users.	2.86 ± 0.90
I believe that workers’ actions and attitudes are governed/protected by professional codes of ethics and the guidelines of Primary Care.	3.16 ± 0.83
In my work, I interact with professionals who demonstrate ideas or attitudes that promote harmony in the workplace.	3.01 ± 0.85
Total Dimension Score (Mean: 3.14 ± 0.59)	47.2 ± 8.86
**Workplace safety**	
In my work, I am able to organize my time and preserve my physical needs (e.g., rest, food and hydration).	2.60 ± 1.04
The physical structure of my workplace is conducive to professional practice.	2.44 ± 1.25
The conditions my job provides make me feel safe and protected.	2.26 ± 1.18
I work in an environment free from physical violence against the worker.	2.52 ± 1.30
I work in an environment free from verbal violence against the worker.	2.19 ± 1.21
I work in an environment free from harassment^*^ against the worker.	2.58 ± 1.22
Total Dimension Score (Mean: 2.43 ± 0.94)	14.6 ± 5.66
**Strategic management for healthy work**	
In my work, there are strategies in place to address worker health.	2.02 ± 1.25
In my workplace, there is an openness for employees to express their feelings, needs, and ethical values.	2.58 ± 1.12
The material resources available at my workplace are sufficient and adequate for carrying out my activities.	2.64 ± 0.94
In my work environment, there is adequate provision and participation of employees in continuing education programs.	2.94 ± 0.94
In my workplace, there are care protocols in place that benefit both my work and that of my colleagues.	3.00 ± 0.97
In my workplace, there are appropriate interventions in place to protect workers from physical hazards (e.g., noise, temperature, dust, vibrations, gases, radiation, light).	2.21 ± 1.20
In my workplace, there are appropriate interventions in place to protect workers from biological hazards (bacteria, viruses, fungi, parasites).	2.67 ± 1.17
In my workplace, there are appropriate interventions in place to protect workers from mechanical loads (which generate risks of fractures, back pain, limb injuries, among others).	2.10 ± 1.22
In my workplace, there are appropriate interventions in place to protect workers from psychological burdens (which generate stress, mental illness, overload, among others).	1.77 ± 1.26
The career plan is known and discussed among colleagues.	1.94 ± 1.24
I feel that my labor rights are not at risk in my work environment (e.g., no benefits being cut, vacations being suspended, etc.).	2.56 ± 1.34
I identify actions to prevent violence against workers and promote a culture of peace (e.g., nonviolent communication) in my workplace.	2.61 ± 1.16
In my workplace, I identify concrete policies and actions aimed at the safety and protection of workers.	2.14 ± 1.24
In my department, there are initiatives to promote a healthy work environment.	2.35 ± 1.20
In my department, employees can have flexible hours without any loss of pay.	2.75 ± 1.23
Total Dimension Score (Mean: 2.42 ± 0.8)	36.3 ± 12.0
**Overall Total Score** (Average: 2.72 ± 0.62)	98.1 ± 22.6

^*^SD = Standard deviation.

In Table 3, the correlation analysis showed that age (r = –0.203; p = 0.001) and length of service at the institution (r_s_ = –0.248; p < 0.001) and time in PHC (r_s_ = –0.177; p = 0.006) showed negative and statistically significant correlations with job safety. Furthermore, time spent at the institution was negatively correlated with strategic management (r_s_ = –0.161; p = 0.012), and time spent in PHC showed a similar correlation (r_s_ = –0.195; p = 0.002). Income showed a weak negative correlation, statistically significant, with motivation for work (r_s_ = –0.142; p = 0.027), indicating that salaries do not necessarily translate into greater motivation. In the comparison of means, the type of temporary employment contract stood out as the only categorical variable with a significant difference, specifically in the strategic management for healthy work dimension (p = 0.047), which shows that nurses with temporary employment contracts evaluated the perception of strategic management for healthy work more positively.

**Table 3:** Analysis of correlations and associations between sociodemographic variables and dimensions of the assessment scales in the workplace of nurses in primary health care – Manaus, AM, Brazil, 2024.

Variables	Motivation for work	Workplace safety	Strategic management for healthy work	Total score
**Continuous variables**				
Age (years) – r^*^	0.059 (p = 0.356)	**–0.203 (p = 0.001)**	**–0.132 (p = 0.040)**	–0.098 (p = 0.128)
Time since graduation (years) – r_s**_	0.061 (p = 0.345)	**–0.162 (p = 0.011)**	–0.122 (p = 0.057)	–0.100 (p = 0.121)
Educational level – r_s**_	0.052 (p = 0.421)	–0.037 (p = 0.565)	0.027 (p = 0.678)	0.025 (p = 0.697)
Income – r_s**_	**–0.142 (p = 0.027)**	–0.075 (p = 0.243)	–0.082 (p = 0.202)	–0.122 (p = 0.057)
Length of service in Primary Care (years) – r_s**_	0.001 (p = 0.984)	**–0.177 (p = 0.006)**	**–0.195 (p = 0.002)**	**–0.159 (p = 0.013)**
Length of service at the current institution (years) – r_s**_	0.059 (p = 0.364)	**–0.248 (p < 0.001)**	**–0.161 (p = 0.012)**	**–0.141 (p = 0.028)**
Weekly workload – r^*^	0.115 (p = 0.074)	–0.112 (p = 0.080)	0.021 (p = 0.747)	0.012 (p = 0.847)
**Categorical variables**	**Mean ± SD**	**Mean ± SD**	**Mean ± SD**	**Mean ± SD**
Sex				
Male	49.0 ± 5.7	15.6 ± 5.1	39.2 ± 11.3	103.7 ± 17.2
Female	47.0 ± 9.2	14.4 ± 5.7	35.9 ± 12.1	97.3 ± 23.2
p value	0.105	0.278	0.154	0.138
Marital status				
Single	45.7 ± 10.1	15.0 ± 5.7	37.1 ± 11.9	97.9 ± 24.2
Married/Common law marriage	47.5 ± 8.1	14.1 ± 5.7	35.4 ± 12.0	97.0 ± 21.9
Divorced	50.0 ± 8.0	15.6 ± 5.3	38.4 ± 13.0	104 ± 21.4
Widower	54.0 ± 2.8	18.0 ± 4.2	37.0 ± 2.8	109 ± 4.2
p value	0.093	0.364	0.579	0.462
Training				
Undergraduate course	47.2 ± 7.3	15.2 ± 6.4	31.8 ± 12.4	94.2 ± 21.2
Residence	43.3 ± 10.1	14.5 ± 5.3	34.9 ± 9.1	92.7 ± 21.6
Graduate certificate course	47.6 ± 8.8	14.7 ± 5.7	36.8 ± 12.1	99.1 ± 22.7
Master’s/Doctorate	46.2 ± 8.9	13.2 ± 6.0	33.8 ± 12.7	93.2 ± 23.9
p value	0.310	0.792	0.462	0.530
Type of relationship				
CLT (Consolidation of Labor Laws – a decree which governs labor relations in Brazil)	45.1 ± 10.5	15.0 ± 6.0	**35.5 ± 13.3^ab^**	95.6 ± 27.2
Permanent contract	46.1 ± 7.5	14.4 ± 5.6	**34.4 ± 12.3^a^**	94.9 ± 21.1
Temporary	48.6 ± 9.7	14.7 ± 5.7	**38.3 ± 11.3^b^**	101.6 ± 23.1
p value	0.073	0.878	**0.047**	0.075
District				
East	48.7 ± 9.0	14.8 ± 5.4	35.7 ± 11.6	99.1 ± 21.1
West	47.7 ± 8.0	16.4 ± 4.5	38.4 ± 10.7	102.5 ± 19.7
North	46.5 ± 8.6	13.6 ± 5.7	35.5 ± 11.6	95.6 ± 21.9
South	46.1 ± 9.7	13.8 ± 6.5	36.0 ± 14.1	95.8 ± 27.0
Rural	47.6 ± 10.2	14.8 ± 5.8	35.4 ± 13.3	97.7 ± 24.6
p value	0.547	0.070	0.708	0.480
Health team that works				
Direction	47.1 ± 10.0	13.6 ± 7.1	37.0 ± 14.7	97.8 ± 28.1
PHC team	46.1 ± 7.8	15.6 ± 5.8	36.3 ± 11.6	97.9 ± 20.8
FHS team	47.7 ± 9.2	14.3 ± 5.5	36.3 ± 12.1	98.2 ± 23.1
p value	0.477	0.258	0.986	0.995
Holds a management position in the PHC – n (%)				
Yes	48.4 ± 7.7	13.7 ± 5.5	37.6 ± 10.1	99.7 ± 19.1
No	47.2 ± 8.5	14.9 ± 5.6	36.4 ± 12.0	98.4 ± 22.2
Not at the moment, but I have occupied it before.	44.6 ± 13.7	14.7 ± 6.8	32.2 ± 16.2	91.5 ± 32.7
p value	0.256	0.445	0.231	0.365

*r = Pearson coefficient;

**r_s_ = Spearman coefficient.

## DISCUSSION

The predominance of women as participants (87.2%) is a finding widely recognized in the literature on the profile of Nursing in Brazil and worldwide. Specialization reflects a high level of qualification among PHC nurses, which aligns with the growing demands in the context of PHC. However, the low proportion of professionals with master’s and doctoral degrees (6.2% each) may indicate gaps in the pursuit of and access to graduate education, which should still be encouraged in professional development policies, addressing the regional imbalance in supply in Brazil. Furthermore, the predominance of temporary contracts indicates an increase in this type of hiring across various healthcare levels and sectors^(16,17)^.

The results revealed that, despite a good level of motivation for work, PHC nurses in Manaus perceive a work environment with weaknesses, especially regarding Workplace Safety and Strategic Management for Healthy Work, corroborating international studies^(18,19)^.

In the Motivation for Work dimension, the study identified a relatively positive score, but there are aspects that suggest weaknesses, with the lowest average in the item “integration, participation, and harmony in the work environment,” which suggests difficulties in integration among colleagues or with management. This may be related to work overload or a lack of institutional actions to strengthen team cohesion^(20)^.

The motivation of nurses, especially in PHC, is a factor that impacts productivity and quality of care. When motivated, these professionals tend to be more engaged in their activities, which translates into better performance and more positive results for users. A finding in Japan, also from research conducted with nurses, highlighted that this factor acts as a catalyst for job engagement, since motivated professionals understand the importance of their role, feel valued, and work in a collaborative environment, as well as tending not to abandon the profession^(20,21)^.

Regarding the analysis of the Motivation for Work dimension, in this sample of nurses who receive an average salary above the Brazilian national average, which is around 3 and a half minimum wages^(22)^, Motivation showed a negative correlation with income, which indicates that motivation does not necessarily increase with an increase in salary. It is important to consider that many nurses feel their salaries do not adequately reflect the workload and responsibility that the job requires^(23)^.

Devaluation of wages results in the perception of devaluation of the profession and can lead to demotivation, decreasing engagement at work. A qualitative study conducted in 23 municipalities located in the state of Minas Gerais also revealed the disparity between salary expectations and the reality faced by professionals, which they indicated is a key factor in dissatisfaction and declining motivation^(24)^. The incomemotivation relationship indicates that, although remuneration is a right, policies that value employees should go beyond salary, investing in recognition, autonomy, and working conditions^(6)^.

In the Workplace Safety dimension, the perception of insecurity was evident, with a low average score, highlighting the areas of physical infrastructure. Researchers argue that inadequate infrastructure not only increases stress among healthcare professionals but also hinders the practice of safe and effective work^(4)^. Environments with poor facilities and the deficit or lack of maintenance or inadequate facilities and equipment at PHC units hinder nurses’ satisfactory performance, a necessary factor for a healthy work environment, which increases the incidence of errors and impairs patient care^(25)^.

According to a study conducted in 2024, improvements in the infrastructure of PHC units, combined with other interventions, contribute to the well-being and quality of life of nurses, with a positive impact on their physical and mental health in the primary health care work environment^(7)^. It is worth highlighting that inadequate infrastructure represents a weakness in worker safety, an operational risk, and a cost to the system that should be a management priority with specific budget allocation^(26)^.

The weak negative correlation between age and perception of workplace safety suggests that more experienced professionals may have a more critical view of their perception of exposure to precarious working conditions and inadequate physical environments, which can expose them to risks of workplace accidents, occupational diseases, and contamination. Studies^(27,28)^ converge in this sense and indicate that more experienced professionals perceive more risks and vulnerabilities in the work environment, which can be explained by the accumulation of experiences and greater awareness of the challenges faced in the PHC daily routine. Integrating the risk perception of experienced nurses into strategic planning is an effective way to anticipate problems, improve safety protocols, increase the confidence of the entire team, and create a healthier and safer work environment for nurses^(27)^.

In this study, exposure to verbal and physical violence emerges as a significant outcome in PHC. Studies indicate that workplace violence is frequently associated with conflicts with clients, work overload, and a lack of protective measures^(5)^. The lack of adequate training to deal with situations of aggression and the absence of institutional support exacerbate the feeling of insecurity among healthcare professionals, harm nurses’ wellbeing, and also create a cycle of dissatisfaction^(7,29)^.

A mixed-methods study, conducted with nurses from both hospital and PHC settings, demonstrates that implementing collaborative communication protocols and creating peer support networks can be effective strategies for reducing violence against nurses in PHC, going beyond individual training and traditional institutional support. Research shows that strengthening interprofessional relationships and building a work environment based on solidarity and mutual support among colleagues significantly contribute to reducing the vulnerability of professionals and to nurses’ mental health^(5)^.

The findings in the Strategic Management for Healthy Work dimension highlight significant gaps in institutional practices for worker care. One of the critical points is the inadequacy of interventions against psychological burdens. This relationship is found in the context of work overload and stress, situations that are frequent in PHC, especially among nurses, with a negative impact on mental health and performance. The lack of adequate strategies to deal with these issues creates an adverse cycle and results in exhausted professionals with sleep disorders and social dysfunctions^(30,31)^. Some measures should be implemented, such as changes in work policies, reduced workload and/or flexible hours, improvements in relationships between colleagues and management teams, and the establishment of intra-institutional mental health counseling centers/points. These are strategies aimed at creating a healthier work environment, strengthening the resilience of nurses and mitigating the negative effects of psychological burdens^(31)^.

The Strategic Management for Healthy Work dimension was shown to be associated with the type of employment contract. Workers with a contract show better insights into Strategic Management, especially temporary workers. This association may reflect differences in expectations between temporary and permanent employees, as well as a possible reluctance to critically evaluate management, despite the guarantee of anonymity in responses, due to their fixed-term contracts; thus, their evaluations may be influenced by the need to preserve long-term relationships within the institution^(32)^.

Temporary hiring, while sometimes necessary to fill gaps in regions lacking nurses, compromises long-term stability and the retention of professionals. This scenario calls for policies that prioritize stable employment relationships, especially in specific areas of Brazil, to ensure a skilled and motivated workforce. The literature suggests investing in public hiring processes and retaining professionals, promoting higher quality in the continuity of care^(16,17)^.

The overall score of WES-PHC provided a regular overall assessment of the work environment. Although nurses demonstrate motivation in their work, the dimensions related to safety and strategic management indicate areas that require attention and improvement to ensure a healthier and safer work environment. These objective and subjective dimensions are fundamental to ensuring the quality of nurses’ professional practices in PHC, with interfaces in a positive work environment capable of promoting the health of nurses and an institutional climate conducive to quality care^(10,11,12)^. It is also important to consider that the practices and working conditions of nursing professionals can vary widely between different primary health care units due to variations in local policies, infrastructure, and management^(33)^.

One limitation to consider is that, although stratification by districts was performed, randomization was not applied within each stratum. This aspect may have influenced the results, as the selection of participants may not have reflected the diversity present in each district in a balanced way, which could affect the generalizability of the findings. Furthermore, more than half of the participating professionals were hired on a temporary basis, which may influence the results in one of the domains assessed by the instrument (strategic management for healthy work), considering the specificities and possible instabilities associated with this type of employment relationship.

## CONCLUSION

The findings of this study revealed a generally average/moderate perception of the work environment among primary health care nurses in Manaus, Brazil. Although they exhibit a good level of motivation at work, aspects such as workplace safety and strategic management for healthy work were negatively evaluated. Furthermore, inferential analyses demonstrated significant associations between sociodemographic characteristics and the dimensions of the WES-PHC scale, indicating that individual and organizational factors can influence the perception and meanings attributed to the work environment. The results highlight the need for interventions focused on improving safety and strategic management, aiming to promote a more favorable environment for professional practice in primary health care.

Challenges include a lack of integration among colleagues and organizational communication, a perception of insecurity in the work environment reflected in the evaluation of the physical structure and protection against violence, highlighting the urgent need for improvements in work environments. Moreover, strategic management for healthy work is still deficient, with a lack of some institutional initiatives aimed at improving the work environment in primary health care. Strategies such as investments in infrastructure, strengthening participatory management, and expanding actions to protect worker health can help minimize negative impacts. In addition, it is essential to promote professional development, with incentives for qualifications, adequate salaries, and job stability.

## Data Availability

The entire dataset supporting the results of this study was published in the article itself.
