# Supplementary material for: Work environment of nurses in primary health care in Manaus: an analytical cross-sectional study
Source: Rev Esc Enferm USP. 2026 Mar 6;60:e20250280. doi: 10.1590/1980-220X-REEUSP-2025-0280en (PMC12965719; doi:10.1590/1980-220X-REEUSP-2025-0280en)
Supplement: Translation

# Translation: Ambiente de trabalho de enfermeiros na atenção primária à saúde de Manaus: estudo transversal analítico*

**DOI:** 10.1590/1980-220X-REEUSP-2025-0280pt

**Authors:** Lucas Lorran Costa de Andrade, Laura Cavalcanti de Farias Brehmer, Elisiane Lorenzini, Sabrina Blasius Faust, Wagner Ferreira Monteiro, Letícia de Lima Trindade, Ronilson Ferreira Freitas, Flávia Regina Souza Ramos

**Affiliations:** 1 Universidade Federal de Santa Catarina, Departamento de Enfermagem, Florianópolis, SC, Brasil.; 2 Universidade Federal do Amazonas, Departamento de Saúde Coletiva, Manaus, AM, Brasil.; 3 Universidade do Estado do Amazonas, Departamento de Enfermagem, Manaus, AM, Brasil.; 4 Universidade do Estado de Santa Catarina, Departamento de Enfermagem, Chapecó, SC, Brasil.

**Keywords:** Atenção Primária à Saúde, Trabalho, Ambiente de Instituições de Saúde, Enfermeiras e Enfermeiros

## Abstract

**Objetivo:**

Analisar as dimensões do ambiente de trabalho dos enfermeiros da Atenção Primária à Saúde e sua relação com características sociodemográficas dos profissionais.

**Método:**

Estudo transversal, analítico, realizado entre abril/outubro de 2024, com 243 enfermeiros da APS de Manaus. Utilizou-se questionário e a Escala de Avaliação do Ambiente de Trabalho. Foi realizada análise descritiva e inferencial.

**Resultados:**

A avaliação global do ambiente de trabalho foi regular com média 98,1. A média da dimensão Motivação para o trabalho foi 47,2. A dimensão Segurança no Ambiente de Trabalho apresentou média mais baixa (14,6), classificada como “atenção”, e Gestão Estratégica para o Trabalho Saudável, com média 36,3, foi classificada com pontos de alerta. Evidenciou-se correlação negativa entre renda e motivação, tempo de atuação na APS com gestão, idade/tempo de atuação e segurança. Enfermeiros temporários avaliaram mais positivamente a gestão estratégica.

**Conclusão:**

Identificaram-se fragilidades na segurança e gestão estratégica, com necessidade de melhorias na infraestrutura, gestão participativa e proteção à saúde dos profissionais para promover um ambiente de trabalho mais seguro e saudável.

## INTRODUÇÃO

A Atenção Primária à Saúde (APS), também reconhecida como Atenção Básica no Brasil, é considerada a principal porta de entrada do Sistema Único de Saúde (SUS) e desempenha papel central na promoção da saúde, na prevenção de doenças e nos cuidados contínuos da população. Este nível assistencial tem se consolidado como eixo central nas políticas de saúde, o que torna fundamental analisar os atores, cenários e o modo como a complexidade das demandas do ambiente de trabalho pode afetar a saúde e o bem-estar dos profissionais, entre os quais os de enfermagem^(1,2)^.

Os enfermeiros nos serviços da APS assumem responsabilidades distintas, incluindo a coordenação de cuidados, gestão de doenças crônicas, educação em saúde, atividades clínicas e administrativas, além de realizarem visitas domiciliares como parte integral de suas funções no atendimento oferecido nas unidades de saúde^(3)^. Apesar das extensas responsabilidades e da importância da atuação dos enfermeiros na APS, a realidade do trabalho desses profissionais é marcada por desafios que podem comprometer a qualidade do cuidado, como sobrecarga, falta de recursos e infraestrutura inadequada, violência no trabalho, o que desencadeia efeitos na saúde física, mental, gerando, inclusive, sofrimento moral^(4,5)^.

Em determinadas regiões, esses desafios podem ser ainda mais acentuados devido às especificidades do contexto local, como a grande extensão territorial, a diversidade cultural e as desigualdades sociais^(6,7)^. No cenário amazônico brasileiro, embora se registrem avanços no planejamento e expansão da APS, os profissionais de saúde atuantes, especialmente enfermeiros, ainda sofrem com a sobrecarga de trabalho. O apoio para superar as condições de trabalho é um obstáculo significativo, bem como a adaptação do ambiente de trabalho dos profissionais de saúde para que compreendam as particularidades do território^(8)^.

Diante desse contexto desafiador, a construção de um ambiente de trabalho saudável (ATS) emerge como uma necessidade fundamental para bem-estar físico e emocional dos enfermeiros. Segundo a Organização Mundial da Saúde (OMS), um ATS é aquele em que trabalhadores e gestores colaboram para promover e proteger a saúde, segurança e bem-estar de todos, além de garantir a sustentabilidade do ambiente de trabalho^(9)^.

Pesquisadores neste campo de ATS enfatizam a importância de considerar tanto as dimensões objetivas, como o ambiente físico e as condições laborais, quanto as dimensões subjetivas, que incluem fatores simbólicos e éticos^(10)^. Ou seja, ao proporcionar condições físicas adequadas, reconhecimento profissional, apoio institucional, oportunidades de desenvolvimento e um clima organizacional positivo, é possível promover a saúde física, mental e social dos enfermeiros. Essa medida contribui para a melhoria da qualidade de vida desses profissionais e impacta positivamente a qualidade da assistência prestada à população^(11)^.

A partir dos estudos realizados sobre a avaliação dos ambientes de trabalho na APS, a utilização da Escala de Avaliação de Ambiente de Trabalho (EAAT-APS) apresenta eixos importantes para o entendimento do impacto do ambiente de trabalho na saúde e no bem-estar dos trabalhadores. A utilização desse instrumento auxilia na identificação de áreas críticas e na tomada de decisões informadas sobre mudanças no ambiente de trabalho, visando a melhoria da qualidade de vida dos trabalhadores e o aumento da produtividade e eficiência das organizações^(10,12)^.

Embora a literatura aponte para os desafios enfrentados por enfermeiros na APS, a grande maioria desses estudos está limitada às regiões Sul e Sudeste do Brasil e ao setor hospitalar^(13,14)^. Esse panorama cria um viés de compreensão, ampliando a lacuna em relação a regiões com contextos singulares, como a Norte. Nesta região, os fatores locais, sejam logísticos, culturais ou de recursos, podem repercutir no ambiente de trabalho. Dessa forma, percebe-se que são escassas as pesquisas que, partindo das particularidades da Amazônia, buscam compreender as percepções dos enfermeiros e identificar os elementos contextuais que moldam seu cotidiano profissional^(15)^.

Diante da complexidade apresentada e da necessidade de aprofundamento do conhecimento, o objetivo do estudo é analisar as dimensões do ambiente de trabalho dos enfermeiros da Atenção Primária à Saúde e sua relação com as características sociodemográficas dos profissionais.

## MÉTODO

### Tipo ou Desenho do Estudo

Trata-se de um estudo transversal analítico, realizado no período de abril a outubro de 2024, relatado conforme as recomendações do guia *Strengthening the Reporting of Observational Studies in Epidemiology* (STROBE).

### Local, População e Critérios de Seleção

A pesquisa foi realizada com enfermeiros da APS do município de Manaus, capital do Estado do Amazonas, que é administrativamente organizado em cinco Distritos de Saúde (DISA): Norte, Leste, Oeste, Sul e Rural, com um total de 288 Unidades Básicas de Saúde (UBS) e 500 profissionais enfermeiros, conforme dados do Cadastro Nacional de Estabelecimentos de Saúde (Consultado no dia 05/02/2024). Foi considerada população elegível a de todos os trabalhadores enfermeiros que estavam em atividades laborais nas UBS no período da coleta de dados, há no mínimo três meses. Foram excluídos enfermeiros que ocupavam exclusivamente cargos administrativos ou de gestão fora das UBS e os afastados por qualquer motivo no período da coleta de dados.

### Definição da Amostra

Foi empregada a amostragem estratificada proporcional, assegurando que o número de enfermeiros participantes representasse adequadamente cada distrito de saúde (Norte, Leste, Oeste, Sul e Rural). A população-alvo foi composta pelos 500 enfermeiros da APS, distribuídos da seguinte forma: Distrito Norte com 140 enfermeiros (28%), Distrito Leste com 121 (24,2%), Distrito Oeste com 106 (21,2%), Distrito Sul com 108 (21,6%) e Distrito Rural com 25 (5%).

O cálculo amostral foi realizado por meio do sistema SestatNet^®^, utilizando os seguintes parâmetros: erro amostral de 5%, nível de confiança de 95% e margem de perda de 10%. Com base nesses critérios, o tamanho da amostra foi definido em 239 enfermeiros, distribuídos proporcionalmente entre os distritos, sendo: 67 enfermeiros do Distrito Norte, 58 do Distrito Leste, 51 do Distrito Oeste, 52 do Distrito Sul e 11 do Distrito Rural. Esse processo de estratificação garantiu que a amostra refletisse fielmente as proporções da população original.

### Coleta de Dados

A coleta de dados foi realizada por meio da aplicação de um questionário elaborado pelos autores composto por questões socioprofissionais, com as seguintes variáveis: idade, sexo (atribuído no nascimento), estado civil, instituição em que trabalha, carga horária semanal, vínculo empregatício, tempo de conclusão da graduação, tempo de experiência na APS, outros vínculos de trabalho, atuação na gestão e formação profissional. Além disso, foi utilizada a Escala de Avaliação do Ambiente de Trabalho na Atenção Primária à Saúde (EAAT-APS) criada e validada no Brasil, que adotou as melhores práticas definidas pela *American Educational Research Association* (AERA) e obteve validade de estrutura interna e confiabilidade^(10,12)^. A escala é composta por 36 itens, distribuídos em três dimensões, as quais refletem as percepções ou sentimentos dos profissionais em seu ambiente de trabalho: dimensão Motivação para o trabalho (15 questões); dimensão Segurança no ambiente de trabalho (6 questões); e dimensão Gestão estratégica para o trabalho saudável (15 questões). Para cada item, o participante assinala a frequência em que a situação/percepção/sentimento ocorre numa progressão de 0 a 4, no qual 0 significa nunca e 4 significa sempre.

Para avaliação do ambiente de trabalhos dos enfermeiros por meio da EAAT-APS, consideraram-se os valores das médias dos resultados na pontuação geral e nas três dimensões: motivação para o trabalho, segurança no ambiente de trabalho e gestão estratégica para o trabalho saudável. Cada dimensão apresenta quatro faixas de classificação: crítica (condição preocupante, com pontuação próxima de zero), ameaça (risco moderado, exigindo melhorias), atenção (pontos de alerta, mas não críticos) e saudável (ambiente adequado e positivo). Na dimensão motivação para o trabalho e na gestão estratégica, os escores variam de 0 a 60, sendo considerados saudáveis quando iguais ou superiores a 45, atenção de 30 a 44, ameaça de 15 a 29 e crítico abaixo de 15. Na dimensão segurança no ambiente de trabalho, os escores vão de 0 a 24, sendo saudável a partir de 18, atenção entre 12 e 17, ameaça de 6 a 11 e crítico abaixo de 6. A pontuação geral varia de 0 a 144, classificada como saudável a partir de 108, atenção entre 72 e 107, ameaça de 36 a 71 e crítico abaixo de 36^(10,12)^.

Os participantes foram inicialmente contatados pelo aplicativo WhatsApp^®^, com os números disponibilizados pelos supervisores ou diretores dos distritos. Por meio desse contato, receberam um link para leitura e aceite do Termo de Consentimento Livre e Esclarecido, e o questionário disponibilizado via *Google Forms.* Em alguns casos, foi necessário realizar a coleta presencialmente com os participantes, agendando um encontro na UBS em um horário conveniente para o profissional, de forma a não interferir em suas atividades.

### Análise e Tratamento dos Dados

O software *Statistical Package for Social Science* (SPSS), versão 23, foi utilizado para a realização das análises. Inicialmente, as variáveis categóricas foram apresentadas em frequências absolutas (n) e relativas (%). As variáveis contínuas foram descritas por meio de médias, desvio padrão (DP), medianas e intervalos interquartis (P25–P75), de acordo com a distribuição dos dados.

A normalidade dos dados em cada variável foi avaliada por meio do teste de Shapiro-Wilk. Para análise da qualidade do ambiente de trabalho segundo as três dimensões da EAATAPS (Motivação para o Trabalho, Segurança no Ambiente de Trabalho e Gestão Estratégica para o Trabalho Saudável) foram calculados média e desvio padrão. Utilizou-se o teste qui-quadrado de *Pearson* para analisar associações entre variáveis categóricas (características sociodemográficas e percepções relacionadas ao ambiente de trabalho) e a análise de correlação de *Spearman* para identificar possíveis correlações entre variáveis contínuas (idade, tempo de formação, tempo de atuação) e as dimensões da EAAT-APS. Ainda se utilizou a Análise de Variância (ANOVA) para comparar médias entre três ou mais grupos, como estado civil, formação, distrito de saúde e outros. Os resultados foram organizados em tabelas, destacando-se as associações significativas considerando p < 0,05.

### Aspectos Éticos

A pesquisa foi conduzida em conformidade com as Diretrizes e Normas Regulamentadoras de Pesquisas Envolvendo Seres Humanos, conforme a Resolução nº 466/12 do Conselho Nacional de Saúde, e aprovada pelo Comitê de Ética em Pesquisa da Universidade Federal de Santa Catarina, no ano de 2024, sob o parecer nº 6.735.782. Todos os participantes consentiram formalmente, assinando o Termo de Consentimento Livre e Esclarecido antes de sua participação no estudo.

## RESULTADOS

A amostra do estudo foi composta por 243 enfermeiros da APS, com idade média de 43,8 anos, que possuem, predominantemente, formação no nível de especialização (83,5%). Prevaleceram enfermeiros do sexo feminino (87,2%), casadas ou em união estável (55,1%), com mediana de tempo de formação de 16 anos, que possuem um vínculo empregatício (86%), e prevaleceram os vínculos temporário (47,3%) e estatutário (46,9%). Predominantemente, os enfermeiros possuíam carga horária semanal de 40 horas (70,8%), e a mediana de tempo de atuação na instituição atual foi de quatro anos. Em relação à renda, o maior percentual de participantes (49%) recebia entre cinco e seis salários-mínimos, conforme apresentado na Tabela 1.

**Tabela 1:** Caracterização sociodemográfica e profissional dos enfermeiros da atenção primária à saúde – Manaus, AM, Brasil, 2024.

Variáveis	n (%)
Idade (anos) – média ± DP^*^	43,8 ± 9,7
Sexo – n (%)	
Masculino	31 (12,8)
Feminino	212 (87,2)
Estado Civil – n (%)	
Solteiro	81 (33,3)
Casado/União estável	134 (55,1)
Divorciado	26 (10,7)
Viúvo	2 (0,8)
Tempo de formação (anos) – mediana (P25–P75)	16 (10–20)
Formação – n (%)	
Graduação	10 (4,1)
Residência	15 (6,2)
Especialização	203 (83,5)
Mestrado/Doutorado	15 (6,2)
Número de vínculos empregatício – n (%)	
Um	209 (86,0)
Dois	34 (14,0)
Tipo de vínculo – n (%)	
CLT	14 (5,8)
Estatutário	114 (46,9)
Temporário	115 (47,3)
Renda – n (%)	
1 a 2 salários-mínimos	2 (0,8)
3 a 4 salários-mínimos	32 (13,2)
5 a 6 salários-mínimos	119 (49,0)
7 a 8 salários-mínimos	68 (28,0)
9 ou mais salários-mínimos	22 (9,1)
Tempo de atuação na Atenção Primária (anos) – mediana (P25–P75)	6 (3–18)
Distrito – n (%)	
Leste	58 (23,9)
Oeste	54 (22,2)
Norte	67 (27,6)
Sul	53 (21,8)
Rural	11 (4,5)
Tempo de atuação na instituição atual (anos) – mediana (P25–P75)	4 (1–6)
Carga horária semanal – n (%)	
20 h	3 (1,2)
30 h	62 (25,5)
40 h	172 (70,8)
60 h	6 (2,5)
Equipe de saúde em que trabalha – n (%)	
Direção	8 (3,3)
EAP	65 (26,7)
ESF	170 (70,0)
Ocupa cargo de gestão na AP – n (%)	
Sim	55 (22,6)
Não	168 (69,1)
No momento não, mas já ocupei	20 (8,2)

^*^DP = desvio padrão.

Os resultados evidenciaram que a dimensão Motivação para o Trabalho apresentou os melhores indicadores, com escore total de 47,2 (±8,86) e média de 3,14 por item, o que reflete uma percepção geral positiva entre os enfermeiros, quanto à motivação. Em contraste, a Segurança no Ambiente de Trabalho foi classificado como “atenção”, com escore total de 14,6 (±5,66) e média de 2,43 por item, o que mostra que há fragilidades na estrutura física e proteção laboral. A Gestão Estratégica para Trabalho Saudável também mostrou resultados com pontos de alerta, mas não críticos, com 36,3 (±12,0) de escore total e média de 2,42 por item, o que indica deficiências nas políticas de proteção à saúde do trabalhador.

O escore geral combinado das três dimensões foi de 98,1 (±22,6), com média de 2,72 por item, posicionando-se na faixa “que demanda atenção” (73–108) conforme os parâmetros da EAAT-APS. No geral, o ambiente avaliado está próximo do satisfatório, mas expressa ameaças e elementos que requerem atenção e intervenção em duas dimensões. Cada dimensão acompanha tal resultado (atenção) apesar das diferenças identificadas. A Tabela 2 apresenta os resultados das três dimensões fornecidas pela EAAP-APS, com os escores totais de cada dimensão e a percepção geral dos enfermeiros sobre seu ambiente de trabalho.

**Tabela 2:** Resultado da avaliação do ambiente de trabalho na atenção primária à saúde por itens e construto – Manaus, AM, Brasil, 2024.

Itens	Média ± DP^*^
**Motivação para o trabalho**
O que eu faço/pratico no meu trabalho traz sentido para a minha vida.	3,55 ± 0,72
Sinto-me envolvido e motivado quando estou trabalhando.	3,38 ± 0,79
Os colegas que trabalham comigo têm conhecimento e competência coerentes com o trabalho que exercem.	3,14 ± 0,78
No meu local de trabalho, a organização do processo de trabalho acontece de acordo com os objetivos da Atenção Primária em Saúde (APS)	3,12 ± 0,86
Tenho acesso às informações necessárias para a realização das minhas atividades.	3,41 ± 0,77
Existe uma adequada e respeitosa comunicação entre equipe e gestão da Unidade, promovendo a participação nas decisões e organização do trabalho.	3,18 ± 0,96
Meus colegas agem de forma integrada (participativa) no ambiente de trabalho	2,99 ± 0,88
No meu local de trabalho, conseguimos resolver conflitos de modo a não prejudicar os objetivos do trabalho.	3,14 ± 0,80
Existe uma adequada comunicação entre os profissionais do meu local de trabalho e os usuários, favorecendo o desenvolvimento do trabalho.	3,06 ± 0,82
Existe articulação da gestão da Unidade de Saúde ou das instâncias representativas da gestão da Atenção Primária com as associações e outras organizações coletivas dos trabalhadores (sindicato de trabalhadores, conselhos de classe, conselho de saúde).	2,59 ± 1,14
Quando trabalho, sinto-me à vontade e livre de constante observação por parte da gestão, coordenação, colegas ou usuários.	3,15 ± 0,96
Estou satisfeito com a imagem que os usuários têm do meu trabalho.	3,53 ± 0,69
Considero que meus colegas de trabalho são valorizados pelos usuários da unidade de saúde.	2,86 ± 0,90
Considero que as ações e atitudes dos trabalhadores são regidas/protegidas pelos códigos de ética profissional e pelas diretrizes da Atenção Primária.	3,16 ± 0,83
No meu trabalho convivo com profissionais que apresentam ideias ou atitudes que favorecem a harmonia no trabalho.	3,01 ± 0,85
Escore Total da Dimensão (Média: 3,14 ± 0,59)	47,2 ± 8,86
**Segurança no ambiente de trabalho**
No meu trabalho, consigo organizar o tempo e preservar minhas necessidades físicas (exemplo: descanso, alimentação e hidratação).	2,60 ± 1,04
A estrutura física do meu local de trabalho é favorável para a prática profissional.	2,44 ± 1,25
As condições que meu trabalho oferece me fazem sentir seguro(a) e protegido(a).	2,26 ± 1,18
Trabalho num ambiente livre de violência física contra o trabalhador.	2,52 ± 1,30
Trabalho num ambiente livre de violência verbal contra o trabalhador.	2,19 ± 1,21
Trabalho num ambiente livre de assédio^*^ contra o trabalhador.	2,58 ± 1,22
Escore Total da Dimensão (Média: 2,43 ± 0,94)	14,6 ± 5,66
**Gestão estratégica para o trabalho saudável**
No meu trabalho existem estratégias de atenção à saúde do trabalhador.	2,02 ± 1,25
No meu trabalho existe abertura para os trabalhadores expressarem seus sentimentos, necessidades e valores éticos.	2,58 ± 1,12
Os recursos materiais disponíveis no meu local de trabalho são suficientes e adequados para a realização das minhas atividades.	2,64 ± 0,94
No meu ambiente de trabalho existe oferta e participação adequadas dos trabalhadores em ações de educação permanente	2,94 ± 0,94
No meu local de trabalho existem protocolos assistenciais que favorecem minhas atividades e de meus colegas.	3,00 ± 0,97
No meu local de trabalho existem intervenções adequadas para proteção do trabalhador frente às cargas físicas (exemplo: ruído, temperatura, poeira, vibrações, gases, radiações, luminosidade)	2,21 ± 1,20
No meu local de trabalho existem intervenções adequadas para proteção do trabalhador frente às cargas biológicas (bactérias, vírus, fungos, parasitas).	2,67 ± 1,17
No meu local de trabalho existem intervenções adequadas para proteção do trabalhador frente às cargas mecânicas (que geram riscos para fratura, dores de coluna, membros entre outros)	2,10
No meu local de trabalho existem intervenções adequadas para proteção do trabalhador frente às cargas psíquicas (geradoras de estresse, adoecimento psíquico, sobrecarga, entre outras).	1,77 ± 1,26
O plano de carreira é conhecido e discutido entre colegas.	1,94 ± 1,24
Sinto que meus direitos de trabalhador não sofrem risco no meu ambiente de trabalho (exemplo: corte de algum benefício, suspensão de férias, entre outros).	2,56 ± 1,34
Identifico ações de prevenção da violência contra o trabalhador e a promoção de uma cultura de paz (exemplo: comunicação não violenta) no meu local de trabalho.	2,61 ± 1,16
No meu local de trabalho eu identifico políticas e ações concretas direcionadas para a segurança e proteção dos trabalhadores.	2,14 ± 1,24
Na minha unidade existem ações para promover o Ambiente de Trabalho Saudável	2,35 ± 1,20
Na minha unidade é possível trabalhadores terem flexibilidade de horários, sem prejuízos de salário.	2,75 ± 1,23
Escore Total da Dimensão (Média: 2,42 ± 0,8)	36,3 ± 12,0
**Escore Total Geral** (Média: 2,72 ± 0,62)	98,1 ± 22,6

*DP = Desvio Padrão.

Na Tabela 3, na análise de correlação, observou-se que idade (r = –0,203; p = 0,001), tempo de atuação na instituição (r_s_ = –0,248; p < 0,001) e tempo na APS (r_s_ = –0,177; p = 0,006) apresentaram correlações negativas e estatisticamente significativas com segurança no trabalho. Além disso, o tempo na instituição correlacionou-se negativamente com gestão estratégica (r_s_ = –0,161; p = 0,012), e o tempo na APS mostrou correlação similar (r_s_ = –0,195; p = 0,002). A renda apresentou correlação fraca negativa, estatisticamente significativa, com motivação para o trabalho (r_s_ = –0,142; p = 0,027), indicando que os salários não necessariamente se traduzem em maior motivação. Na comparação de médias, o tipo de vínculo empregatício temporário destacou-se como a única variável categórica com diferença significativa, especificamente na dimensão gestão estratégica para trabalho saudável (p = 0,047), o que mostra que enfermeiros com vínculo de trabalho temporário avaliaram mais positivamente a percepção da gestão estratégica para o trabalho saudável.

**Tabela 3:** Análise de correlações e associações entre as variáveis sociodemográficas e as dimensões da escalas de avaliação no ambiente de trabalho de enfermeiros na APS – Manaus, AM, Brasil, 2024.

Variáveis	Motivação para o trabalho	Segurança no ambiente de trabalho	Gestão estratégica para o trabalho saudável	Escore total
**Variáveis contínuas**				
Idade (anos) – r^*^	0,059 (p = 0,356)	**–0,203 (p = 0,001)**	**–0,132 (p = 0,040)**	–0,098 (p = 0,128)
Tempo de formação (anos) – r_s_^**^	0,061 (p = 0,345)	**–0,162 (p = 0,011)**	–0,122 (p = 0,057)	–0,100 (p = 0,121)
Nível de escolaridade – r_s_^**^	0,052 (p = 0,421)	–0,037 (p = 0,565)	0,027 (p = 0,678)	0,025 (p = 0,697)
Renda – r_s_^**^	**–0,142 (p = 0,027)**	–0,075 (p = 0,243)	–0,082 (p = 0,202)	–0,122 (p = 0,057)
Tempo de atuação na Atenção Primária (anos) – r_s_^**^	0,001 (p = 0,984)	**–0,177 (p = 0,006)**	**–0,195 (p = 0,002)**	**–0,159 (p = 0,013)**
Tempo de atuação na instituição atual (anos) – r_s_^**^	0,059 (p = 0,364)	**–0,248 (p < 0,001)**	**–0,161 (p = 0,012)**	**–0,141 (p = 0,028)**
Carga horária semanal – r^*^	0,115 (p = 0,074)	–0,112 (p = 0,080)	0,021 (p = 0,747)	0,012 (p = 0,847)
**Variáveis categóricas**	**Média ± DP**	**Média ± DP**	**Média ± DP**	**Média ± DP**
Sexo				
Masculino	49,0 ± 5,7	15,6 ± 5,1	39,2 ± 11,3	103,7 ± 17,2
Feminino	47,0 ± 9,2	14,4 ± 5,7	35,9 ± 12,1	97,3 ± 23,2
p-valor	0,105	0,278	0,154	0,138
Estado Civil				
Solteiro	45,7 ± 10,1	15,0 ± 5,7	37,1 ± 11,9	97,9 ± 24,2
Casado/União estável	47,5 ± 8,1	14,1 ± 5,7	35,4 ± 12,0	97,0 ± 21,9
Divorciado	50,0 ± 8,0	15,6 ± 5,3	38,4 ± 13,0	104 ± 21,4
Viúvo	54,0 ± 2,8	18,0 ± 4,2	37,0 ± 2,8	109 ± 4,2
p-valor	0,093	0,364	0,579	0,462
Formação				
Graduação	47,2 ± 7,3	15,2 ± 6,4	31,8 ± 12,4	94,2 ± 21,2
Residência	43,3 ± 10,1	14,5 ± 5,3	34,9 ± 9,1	92,7 ± 21,6
Especialização	47,6 ± 8,8	14,7 ± 5,7	36,8 ± 12,1	99,1 ± 22,7
Mestrado/Doutorado	46,2 ± 8,9	13,2 ± 6,0	33,8 ± 12,7	93,2 ± 23,9
p-valor	0,310	0,792	0,462	0,530
Tipo de vínculo				
CLT	45,1 ± 10,5	15,0 ± 6,0	**35,5 ± 13,3^ab^**	95,6 ± 27,2
Estatutário	46,1 ± 7,5	14,4 ± 5,6	**34,4 ± 12,3^a^**	94,9 ± 21,1
Temporário	48,6 ± 9,7	14,7 ± 5,7	**38,3 ± 11,3^b^**	101,6 ± 23,1
p-valor	0,073	0,878	**0,047**	0,075
Distrito				
Leste	48,7 ± 9,0	14,8 ± 5,4	35,7 ± 11,6	99,1 ± 21,1
Oeste	47,7 ± 8,0	16,4 ± 4,5	38,4 ± 10,7	102,5 ± 19,7
Norte	46,5 ± 8,6	13,6 ± 5,7	35,5 ± 11,6	95,6 ± 21,9
Sul	46,1 ± 9,7	13,8 ± 6,5	36,0 ± 14,1	95,8 ± 27,0
Rural	47,6 ± 10,2	14,8 ± 5,8	35,4 ± 13,3	97,7 ± 24,6
p-valor	0,547	0,070	0,708	0,480
Equipe de saúde que trabalha				
Direção	47,1 ± 10,0	13,6 ± 7,1	37,0 ± 14,7	97,8 ± 28,1
EAP	46,1 ± 7,8	15,6 ± 5,8	36,3 ± 11,6	97,9 ± 20,8
ESF	47,7 ± 9,2	14,3 ± 5,5	36,3 ± 12,1	98,2 ± 23,1
p-valor	0,477	0,258	0,986	0,995
Ocupa cargo de gestão na AP – n (%)				
Sim	48,4 ± 7,7	13,7 ± 5,5	37,6 ± 10,1	99,7 ± 19,1
Não	47,2 ± 8,5	14,9 ± 5,6	36,4 ± 12,0	98,4 ± 22,2
No momento não, mas já ocupei	44,6 ± 13,7	14,7 ± 6,8	32,2 ± 16,2	91,5 ± 32,7
p-valor	0,256	0,445	0,231	0,365

^*^r = coeficiente de Pearson

^**^r_s_ = coeficiente de Spearman.

## DISCUSSÃO

O predomínio de mulheres como participantes (87,2%) é um achado amplamente reconhecido na literatura sobre o perfil da Enfermagem no Brasil e no mundo. A especialização reflete um nível elevado de qualificação entre os enfermeiros da APS, o que está alinhado com as exigências crescentes no contexto da atenção à saúde na APS. No entanto, a baixa proporção de profissionais com mestrado e doutorado (6,2% cada) pode indicar lacunas na busca e no acesso à formação *stricto sensu*, que ainda deve ser incentivada em políticas de desenvolvimento profissional, enfrentando o desequilíbrio regional da oferta no Brasil. Além disso, o predomínio de vínculos temporários indica o aumento desse tipo de contratação em diversos níveis e setores da saúde^(16,17)^.

Os resultados revelaram que, apesar de um bom nível de Motivação para o trabalho, os enfermeiros da APS de Manaus percebem um ambiente de trabalho com fragilidades, especialmente no que se refere à Segurança no Ambiente de Trabalho e a Gestão Estratégica para o Trabalho Saudável, corroborando estudos internacionais^(18,19)^.

Na dimensão Motivação para o Trabalho, o estudo identificou uma pontuação relativamente positiva, porém há aspectos que sugerem fragilidades, com a menor média no item “integração, participação e harmonia no ambiente de trabalho”, que sugere dificuldades na integração entre colegas ou com a gestão. Isso pode estar relacionado à sobrecarga de trabalho ou à falta de ações institucionais para fortalecer a união das equipes^(20)^.

A motivação dos enfermeiros, especialmente na APS, é um fator que impacta na produtividade e na qualidade do cuidado. Quando estão motivados, esses profissionais tendem a ser mais engajados em suas atividades, o que se traduz em um melhor desempenho e em resultados mais positivos para os usuários. Uma descoberta no Japão também em pesquisa realizada com enfermeiros destacou que esse fator atua como um catalisador para o engajamento no trabalho, visto que profissionais motivados compreendem a importância de seu papel, sentem-se valorizados e trabalham em um ambiente colaborativo, bem como tendem a não abandonar a profissão^(20,21)^.

Quanto à análise da dimensão Motivação para o trabalho, nesta amostra de enfermeiros que recebem uma média salarial acima da média nacional brasileira, que é em torno de 3 saláriosmínimos e meio^(22)^, a Motivação apresentou correlação negativa com a renda, o que mostra que a motivação não aumenta, necessariamente, com o aumento do salário. É importante considerar que muitos enfermeiros sentem que seus salários não refletem adequadamente a carga de trabalho e a responsabilidade que o perfil do cargo exige^(23)^.

A desvalorização salarial resulta na percepção de desvalorização da profissão e pode levar à desmotivação, diminuindo o engajamento no trabalho. Um estudo qualitativo realizado em 23 municípios localizados no estado de Minas Gerais também apresentou a disparidade entre as expectativas salariais e a realidade enfrentada pelos profissionais, os quais indicaram que é um fator chave para o descontentamento e o declínio na motivação^(24)^. A relação renda-motivação sinaliza que, embora a remuneração seja um direito, políticas de valorização devem ir além do salário, investindo em reconhecimento, autonomia e condições de trabalho^(6)^.

Na dimensão Segurança no Ambiente de Trabalho, a percepção de insegurança foi evidente, com uma pontuação média baixa, com pontos de atenção para os itens de estrutura física. Pesquisadores discutem que infraestruturas inadequadas não só aumentam o estresse dos profissionais de saúde, mas também dificultam a prática de um trabalho seguro e eficaz^(4)^. Ambientes com instalações precárias e o déficit ou a falta de manutenção das UBS e de equipamentos impedem o desempenho satisfatório dos enfermeiros, fator necessário para um ambiente de trabalho saudável, o que eleva a incidência de erros e prejudica o cuidado aos pacientes^(25)^.

De acordo com um estudo realizado em 2024, as melhorias na infraestrutura das UBS, associadas a outras intervenções, contribuem para o bem-estar e a qualidade de vida dos enfermeiros, com impacto positivo na saúde física e mental no ambiente de trabalho na APS^(7)^. Vale ressaltar que a infraestrutura inadequada representa uma fragilidade na segurança do trabalhador, um risco operacional e custo para o sistema que deve ser uma prioridade da gestão com alocação orçamentária específica^(26)^.

A correlação negativa fraca entre a idade e a percepção de Segurança no Ambiente de Trabalho sugere que profissionais com mais experiência podem ter um olhar mais crítico na percepção da exposição às condições de trabalho mais precárias, ambiente físico inadequado, as quais podem expor os profissionais a riscos de acidentes de trabalho, doenças ocupacionais e contaminações. Estudos^(27,28)^ convergem nesse sentido e sinalizam que profissionais mais experientes percebem mais riscos e fragilidades no ambiente de trabalho, o que pode ser explicado pelo acúmulo de vivências e maior consciência dos desafios enfrentados no cotidiano da APS. Integrar a percepção de risco de enfermeiros experientes no planejamento estratégico é uma forma eficaz de antecipar problemas, melhorar os protocolos de segurança, aumentar a confiança de toda a equipe, tornando o ambiente de trabalho mais saudável e seguro para os enfermeiros^(27)^.

Nesse estudo, a exposição à violência verbal e física configura-se como um resultado significativo na APS. Estudos indicam que a violência no ambiente de trabalho está frequentemente associada a conflitos com usuários, sobrecarga de trabalho e falta de medidas de proteção^(5)^. A falta de treinamento adequado para lidar com situações de agressão e a ausência de suporte institucional agravam a sensação de insegurança entre os profissionais de saúde, prejudicam o bem-estar dos enfermeiros e também criam um ciclo de insatisfação^(7,29)^.

Um estudo de abordagem mista, realizado com enfermeiros do contexto hospitalar e da APS, demonstra que a implementação de protocolos de comunicação colaborativa e a criação de redes de apoio entre pares podem ser estratégias eficazes para reduzir a violência contra enfermeiros na APS, indo além do treinamento individual e do suporte institucional tradicional. A pesquisa evidencia que o fortalecimento das relações interprofissionais e a construção de um ambiente de trabalho baseado na solidariedade e no apoio mútuo entre colegas contribuem significativamente para a redução da vulnerabilidade dos profissionais e para a saúde mental dos enfermeiros^(5)^.

Os achados na dimensão Gestão Estratégica para o Trabalho Saudável evidenciam lacunas significativas em práticas institucionais de cuidado com o trabalhador. Um dos pontos críticos é a insuficiência de intervenções contra cargas psíquicas. Essa relação é encontrada no contexto de sobrecarga de trabalho e estresse, situações frequentes na APS, principalmente em enfermeiros, com impacto negativo na saúde mental e no desempenho. A falta de estratégias adequadas para lidar com essas questões cria um ciclo adverso e resulta em profissionais exaustos, com distúrbios do sono e disfunções sociais^(30,31)^. Algumas medidas devem ser implementadas, como mudanças nas políticas de trabalho, redução da carga de trabalho e/ou horários flexíveis, melhorias na relação entre colegas e equipe de gestão e o estabelecimento de centros/pontos de consultoria mental intrainstitucionais. Essas são estratégias que visam criar um ambiente de trabalho mais saudável, fortalecendo a resiliência dos enfermeiros e atenuando efeitos negativos das cargas psíquicas^(31)^.

A dimensão Gestão Estratégica para o Trabalho Saudável mostrou-se associada ao tipo de vínculo empregatício. Os trabalhadores com vínculos apresentam melhores percepções sobre Gestão Estratégica, com destaque para os trabalhadores temporários. Essa associação pode refletir diferenças nas expectativas entre profissionais temporários e estatutários e, também, possível receio de avaliar criticamente a gestão, apesar da garantia do anonimato nas respostas, por estarem em vínculos com prazo determinado; assim, suas avaliações podem ser influenciadas pela necessidade de preservar relacionamentos de longo prazo dentro da instituição^(32)^.

As contratações temporárias, embora às vezes necessárias para cobrir lacunas em regiões carentes de enfermeiros, comprometem a estabilidade de longo prazo e a retenção de profissionais. Esse cenário exige políticas que priorizem vínculos estáveis, especialmente em áreas especificas do Brasil, para assegurar uma força de trabalho qualificada e motivada. A literatura sugere investir em concursos públicos e na fixação de profissionais, promovendo maior qualidade na continuidade do cuidado^(16,17)^.

O escore geral da EAAT-APS mostrou uma avaliação global regular do ambiente de trabalho. Embora os enfermeiros demonstrem motivação com o trabalho, as dimensões relacionadas à segurança e à gestão estratégica indicam áreas que necessitam de atenção e melhorias para garantir um ambiente de trabalho mais saudável e seguro. Essas dimensões objetivas e subjetivas são fundamentais para garantir a qualidade das práticas profissionais dos enfermeiros na APS, com interfaces no clima de trabalho positivo capaz de promover a saúde dos enfermeiros e um clima institucional favorecedor da qualidade dos cuidados^(10,11,12)^. Cabe ainda analisar que as práticas e condições de trabalho dos profissionais de enfermagem podem variar amplamente entre diferentes unidades de APS devido a variações nas políticas locais, infraestrutura e gestão^(33)^.

Uma limitação a considerar é que, embora tenha sido realizada a estratificação por distritos, não foi aplicada aleatorização dentro de cada estrato. Esse aspecto pode ter influenciado os resultados, pois a escolha dos participantes pode não ter refletido de forma equilibrada a diversidade presente em cada distrito, o que pode afetar a generalização dos achados. Além disso, mais da metade dos profissionais participantes foi contratada em regime temporário, podendo influenciar os resultados em um dos domínios avaliados pelo instrumento (gestão estratégica para o trabalho saudável), considerando as especificidades e possíveis instabilidades associadas a esse tipo de vínculo empregatício.

## CONCLUSÃO

Os achados desta pesquisa evidenciaram uma percepção geral regular/moderada do ambiente de trabalho de enfermeiros da APS de Manaus, Brasil. Ainda que apresentem um bom nível de motivação no trabalho, aspectos como a segurança no ambiente laboral e a gestão estratégica para um trabalho saudável foram avaliados negativamente. Além disso, as análises inferenciais demonstraram associações significativas entre características sociodemográficas e as dimensões da escala EAAT-APS, indicando que fatores individuais e organizacionais podem influenciar na percepção e nos significados atribuídos ao ambiente de trabalho. Os resultados destacam a necessidade de intervenções focadas em melhorar a segurança e a gestão estratégica, visando a promoção de um ambiente mais favorável à prática profissional na APS.

Evidenciam-se desafios, como a falta de integração entre colegas e a comunicação organizacional, percepção de insegurança no ambiente de trabalho, refletida na avaliação da estrutura física e da proteção contra violência, com necessidade urgente de melhorias nos ambientes laborais. Além disso, a gestão estratégica para um trabalho saudável ainda se mostra deficiente, com ausência de algumas iniciativas institucionais voltadas à melhoria do ambiente de trabalho na APS. Estratégias como investimentos em infraestrutura, fortalecimento da gestão participativa e ampliação das ações de proteção à saúde do trabalhador podem contribuir para minimizar os impactos negativos. Além disso, é fundamental promover a valorização profissional, com incentivos à qualificação, salários adequados e à estabilidade no emprego.

## Data Availability

Todo o conjunto de dados que dá suporte aos resultados deste estudo foi publicado no próprio artigo.

# Translation: Ambiente de trabajo de enfermeros en atención primaria de salud en Manaus: un estudio transversal analítico*

**DOI:** 10.1590/1980-220X-REEUSP-2025-0280es

**Authors:** Lucas Lorran Costa de Andrade, Laura Cavalcanti de Farias Brehmer, Elisiane Lorenzini, Sabrina Blasius Faust, Wagner Ferreira Monteiro, Letícia de Lima Trindade, Ronilson Ferreira Freitas, Flávia Regina Souza Ramos

**Affiliations:** 1 Universidade Federal de Santa Catarina, Departamento de Enfermagem, Florianópolis, SC, Brasil.; 2 Universidade Federal do Amazonas, Departamento de Saúde Coletiva, Manaus, AM, Brasil.; 3 Universidade do Estado do Amazonas, Departamento de Enfermagem, Manaus, AM, Brasil.; 4 Universidade do Estado de Santa Catarina, Departamento de Enfermagem, Chapecó, SC, Brasil.

**Keywords:** Atención Primaria de Salud, Trabajo, Ambiente de Instituciones de Salud, Enfermeras y Enfermeros

## Abstract

**Objetivo:**

Analizar las dimensiones del ambiente laboral de enfermeros de atención primaria de salud y su relación con las características sociodemográficas de estos profesionales.

**Método:**

Se trata de un estudio transversal, analítico, realizado entre abril y octubre de 2024 con 243 enfermeros de atención primaria de salud en Manaus. Se utilizó un cuestionario y la Escala de Evaluación del Clima Laboral. Se realizó análisis descriptivo e inferencial.

**Resultados:**

La valoración global del ambiente laboral fue media, con una puntuación de 98,1. La puntuación media de la dimensión Motivación para el Trabajo fue de 47,2. La dimensión Seguridad en el Trabajo presentó el promedio más bajo (14,6), clasificada como de “atención”, y la Gestión Estratégica para el Trabajo Saludable, con un promedio de 36,3, fue clasificada como con puntos de alerta. Se encontró una correlación negativa entre los ingresos y la motivación, el tiempo de trabajo en atención primaria de salud con la gerencia, la edad/tiempo de trabajo y la seguridad laboral. Los enfermeros temporales dieron una valoración más positiva de la gestión estratégica.

**Conclusión:**

Se identificaron debilidades en la seguridad y la gestión estratégica, destacando la necesidad de mejoras en la infraestructura, la gestión participativa y la protección de la salud de los profesionales para promover un ambiente de trabajo más seguro y saludable.

## INTRODUCCIÓN

La Atención Primaria de Salud (APS), también conocida como Atención Básica en Brasil, se considera la principal puerta de entrada al Sistema Único de Salud (SUS) y desempeña un papel central en la promoción de la salud, la prevención de enfermedades y la atención continua de la población. Este nivel de asistencia se ha consolidado como eje central de las políticas de salud, lo que hace fundamental analizar los actores, los escenarios y la forma en que la complejidad de las exigencias del entorno laboral puede afectar a la salud y el bienestar de los profesionales, entre ellos los de enfermería^(1,2)^.

Los enfermeros de los servicios de APS asumen diferentes responsabilidades, entre ellas la coordinación de la atención, la gestión de enfermedades crónicas, la educación en salud, las actividades clínicas y administrativas, además de realizar visitas domiciliarias como parte integral de sus funciones en la atención prestada en las unidades de salud^(3)^. A pesar de las amplias responsabilidades y la importancia del papel de los enfermeros en la APS, la realidad laboral de estos profesionales se caracteriza por retos que pueden comprometer la calidad de la atención, como la sobrecarga, la falta de recursos y la infraestructura inadecuada, la violencia en el trabajo, lo que tiene efectos en la salud física y mental, generando incluso sufrimiento moral^(4,5)^.

En determinadas regiones, estos retos pueden ser aún más acusados debido a las especificidades del contexto local, como la gran extensión territorial, la diversidad cultural y las desigualdades sociales^(6,7)^. En el contexto amazónico brasileño, aunque se registran avances en la planificación y expansión de la APS, los profesionales de la salud en activo, especialmente los enfermeros, siguen sufriendo una sobrecarga de trabajo. El apoyo para superar las condiciones de trabajo es un obstáculo significativo, así como la adaptación del entorno de trabajo de los profesionales de la salud para que comprendan las particularidades del territorio^(8)^.

Ante este contexto desafiante, la construcción de un entorno de trabajo saludable (ETS) surge como una necesidad fundamental para el bienestar físico y emocional de los enfermeros. Según la Organización Mundial de la Salud (OMS), un ETS es aquel en el que los trabajadores y los gestores colaboran para promover y proteger la salud, la seguridad y el bienestar de todos, además de garantizar la sostenibilidad del entorno de trabajo^(9)^.

Los investigadores en este campo de los ETS enfatizan la importancia de considerar tanto las dimensiones objetivas, como el entorno físico y las condiciones laborales, como las dimensiones subjetivas, que incluyen factores simbólicos y éticos^(10)^. Es decir, al proporcionar condiciones físicas adecuadas, reconocimiento profesional, apoyo institucional, oportunidades de desarrollo y un clima organizacional positivo, es posible promover la salud física, mental y social de los enfermeros. Esta medida contribuye a mejorar la calidad de vida de estos profesionales y repercute positivamente en la calidad de la asistencia prestada a la población^(11)^.

A partir de los estudios realizados sobre la evaluación de los entornos de trabajo en la APS, la utilización de la Escala de Evaluación del Ambiente de Trabajo (EAAT-APS en la sigla en portugués) presenta ejes importantes para comprender el impacto del entorno de trabajo en la salud y el bienestar de los trabajadores. El uso de este instrumento ayuda a identificar áreas críticas y a tomar decisiones informadas sobre cambios en el entorno laboral, con el objetivo de mejorar la calidad de vida de los trabajadores y aumentar la productividad y la eficiencia de las organizaciones^(10,12)^.

Aunque la literatura señala los retos a los que se enfrentan los enfermeros en la APS, la gran mayoría de estos estudios se limitan a las regiones sur y sureste de Brasil y al sector hospitalario^(13,14)^. Este panorama crea un sesgo de comprensión, ampliando la brecha en relación con regiones con contextos singulares, como el norte. En esta región, los factores locales, ya sean logísticos, culturales o de recursos, pueden repercutir en el entorno laboral. De este modo, se observa que son escasas las investigaciones que, partiendo de las particularidades de la Amazonía, buscan comprender las percepciones de los enfermeros e identificar los elementos contextuales que moldean su día a día profesional^(15)^.

Ante la complejidad presentada y la necesidad de profundizar en el conocimiento, el objetivo del estudio es analizar las dimensiones del entorno laboral de los enfermeros de Atención Primaria de Salud y su relación con las características sociodemográficas de los profesionales.

## MÉTODO

### Tipo o Diseño del Estudio

Se trata de un estudio transversal analítico, realizado entre abril y octubre de 2024, descrito de acuerdo con las recomendaciones de la guía Strengthening the Reporting of Observational Studies in Epidemiology (STROBE).

### Lugar, Población y Criterios de Selección

La investigación se llevó a cabo con enfermeros de APS del municipio de Manaus, capital del estado de Amazonas, que está organizado administrativamente en cinco distritos sanitarios (DISA): Norte, Este, Oeste, Sur y Rural, con un total de 288 Unidades Básicas de Salud (UBS) y 500 profesionales de enfermería, según datos del Registro Nacional de Establecimientos de Salud (consultado el 05/02/2024). Se consideró población elegible a todos los trabajadores de enfermería que estaban trabajando en las UBS durante el periodo de Recolección de datos, desde hacía al menos tres meses. Se excluyó a los enfermeros que ocupaban exclusivamente puestos administrativos o de gestión fuera de las UBS y a los que se encontraban ausentes por cualquier motivo durante el periodo de Recolección de datos.

### Definición de la Muestra

Se utilizó un muestreo estratificado proporcional, asegurando que el número de enfermeros participantes representara adecuadamente a cada distrito sanitario (Norte, Este, Oeste, Sur y Rural). La población objetivo estaba compuesta por 500 enfermeros de la APS, distribuidos de la siguiente manera: Distrito Norte con 140 enfermeros (28 %), Distrito Este con 121 (24,2 %), Distrito Oeste con 106 (21,2 %), Distrito Sur con 108 (21,6 %) y Distrito Rural con 25 (5 %).

El cálculo de la muestra se realizó mediante el sistema SestatNet^®^, utilizando los siguientes parámetros: error muestral del 5 %, nivel de confianza del 95 % y margen de pérdida del 10 %. Basándose en estos criterios, el tamaño de la muestra se fijó en 239 enfermeros, distribuidos proporcionalmente entre los distritos, siendo: 67 enfermeros del Distrito Norte, 58 del Distrito Este, 51 del Distrito Oeste, 52 del Distrito Sur y 11 del Distrito Rural. Este proceso de estratificación garantizó que la muestra reflejara fielmente las proporciones de la población original.

### Recolección de Datos

La recolección de datos se realizó mediante la aplicación de un cuestionario elaborado por los autores, compuesto por preguntas socioprofesionales, con las siguientes variables: edad, sexo (asignado al nacer), estado civil, institución en la que trabaja, carga horaria semanal, vínculo laboral, tiempo de finalización de la licenciatura, tiempo de experiencia en APS, otros vínculos laborales, actuación en la gestión y formación profesional. Además, se utilizó la Escala de Evaluación del Ambiente de Trabajo en la Atención Primaria de Salud (EAAT-APS en la sigla en portugués), creada y validada en Brasil, que adoptó las mejores prácticas definidas por la American Educational Research Association (AERA) y obtuvo validez de estructura interna y confiabilidad^(10,12)^. La escala se compone de 36 ítems, distribuidos en tres dimensiones, que reflejan las percepciones o sentimientos de los profesionales en su entorno laboral: dimensión Motivación para el trabajo (15 preguntas); dimensión Seguridad en el entorno laboral (6 preguntas); y dimensión Gestión estratégica para un trabajo saludable (15 preguntas). Para cada ítem, el participante señala la frecuencia con la que se produce la situación/percepción/sentimiento en una progresión de 0 a 4, en la que 0 significa nunca y 4 significa siempre.

Para evaluar el entorno laboral de los enfermeros mediante la EAAT-APS, se consideraron los valores de las medias de los resultados en la puntuación general y en las tres dimensiones: motivación para el trabajo, seguridad en el entorno laboral y gestión estratégica para un trabajo saludable. Cada dimensión presenta cuatro rangos de clasificación: crítica (condición preocupante, con puntuación cercana a cero), amenaza (riesgo moderado, que requiere mejoras), atención (puntos de alerta, pero no críticos) y saludable (entorno adecuado y positivo). En la dimensión motivación para el trabajo y en la gestión estratégica, las puntuaciones varían de 0 a 60, considerándose saludables cuando son iguales o superiores a 45, atención de 30 a 44, amenaza de 15 a 29 y críticas por debajo de 15. En la dimensión de seguridad en el entorno laboral, las puntuaciones van de 0 a 24, considerándose saludable a partir de 18, atención entre 12 y 17, amenaza de 6 a 11 y crítica por debajo de 6. La puntuación general varía de 0 a 144, clasificándose como saludable a partir de 108, atención entre 72 y 107, amenaza de 36 a 71 y crítica por debajo de 36^(10,12)^.

Inicialmente, se contactó a los participantes a través de la aplicación WhatsApp^®^, con los números proporcionados por los supervisores o directores de los distritos. A través de este contacto, recibieron un enlace para leer y aceptar el Término de Consentimiento Libre y Esclarecido, y el cuestionario disponible a través de Google Forms. En algunos casos, fue necesario realizar la recolección en persona con los participantes, programando una reunión en la UBS en un horario conveniente para el profesional, de manera que no interfiriera en sus actividades.

### Análisis y Tratamiento de los Datos

Se utilizó el software Statistical Package for Social Science (SPSS), versión 23, para realizar los análisis. Inicialmente, las variables categóricas se presentaron en frecuencias absolutas (n) y relativas (%). Las variables continuas se describieron mediante medias, desviación estándar (DE), medianas e intervalos intercuartílicos (P25–P75), de acuerdo con la distribución de los datos.

La normalidad de los datos en cada variable se evaluó mediante la prueba de Shapiro-Wilk. Para analizar la calidad del entorno laboral según las tres dimensiones de la EAAT-APS (motivación para el trabajo, seguridad en el entorno laboral y gestión estratégica para un trabajo saludable), se calcularon la media y la desviación estándar. Se utilizó la prueba Chi-cuadrado de Pearson para analizar las asociaciones entre variables categóricas (características sociodemográficas y percepciones relacionadas con el entorno laboral) y el análisis de correlación de Spearman para identificar posibles correlaciones entre variables continuas (edad, tiempo de formación, tiempo de actuación) y las dimensiones de la EAAT-APS. También se utilizó el análisis de varianza (ANOVA) para comparar medias entre tres o más grupos, como estado civil, formación, distrito sanitario y otros. Los resultados se organizaron en tablas, destacando las asociaciones significativas considerando p < 0,05.

### Aspectos Éticos

La investigación se llevó a cabo de conformidad con las Directrices y Normas Reguladoras de Investigaciones con Seres Humanos, según la Resolución n.º 466/12 del Consejo Nacional de Salud, y fue aprobada por el Comité de Ética en Investigación de la Universidad Federal de Santa Catarina, en el año 2024, bajo el dictamen n.º 6.735.782. Todos los participantes dieron su consentimiento formal, firmando el Término de Consentimiento Libre e Informado antes de su participación en el estudio.

## RESULTADOS

La muestra del estudio estuvo compuesta por 243 enfermeros de APS, con una edad media de 43,8 años, que poseen, predominantemente, formación a nivel de especialización (83,5 %). Predominaban las enfermeras (87,2 %), casadas o en unión libre (55,1 %), con una mediana de tiempo de formación de 16 años, que tenían un vínculo laboral (86 %), y predominaban los vínculos temporales (47,3 %) y estatutarios (46,9 %). Predominantemente, los enfermeros tenían una carga horaria semanal de 40 horas (70,8 %), y la mediana del tiempo de actuación en la institución actual era de cuatro años. En relación con los ingresos, el mayor porcentaje de participantes (49 %) percibía entre cinco y seis salarios mínimos, tal y como se presenta en la Tabla 1.

**Tabla 1:** Caracterización sociodemográfica y profesional de los enfermeros de atención primaria de salud – Manaus, AM, Brasil, 2024.

Variables	n (%)
Edad (años) – media ± DE^*^	43,8 ± 9,7
Sexo – n (%)	
Masculino	31 (12,8)
Femenino	212 (87,2)
Estado civil – n (%)	
Soltero	81 (33,3)
Casado/Unión estable	134 (55,1)
Divorciado	26 (10,7)
Viudo	2 (0,8)
Tiempo de formación (años) – mediana (P25–P75)	16 (10–20)
Formación – n (%)	
Licenciatura	10 (4,1)
Residencia	15 (6,2)
Especialización	203 (83,5)
Máster/Doctorado	15 (6,2)
Número de vínculos laborales – n (%)	
Uno	209 (86,0)
Dos	34 (14,0)
Tipo de vínculo – n (%)	
CLT	14 (5,8)
Estatutario	114 (46,9)
Temporal	115 (47,3)
Ingresos – n (%)	
1 a 2 salarios mínimos	2 (0,8)
3 a 4 salarios mínimos	32 (13,2)
5 a 6 salarios mínimos	119 (49,0)
7 a 8 salarios mínimos	68 (28,0)
9 o más salarios mínimos	22 (9,1)
Tiempo de actuación en Atención Primaria (años) – mediana (P25–P75)	6 (3–18)
Distrito – n (%)	
Este	58 (23,9)
Oeste	54 (22,2)
Norte	67 (27,6)
Sur	53 (21,8)
Rural	11 (4,5)
Tiempo de actuación en la institución actual (años) – mediana (P25–P75)	4 (1–6)
Carga horaria semanal – n (%)	
20	3 (1,2)
30	62 (25,5)
40	172 (70,8)
60 h	6 (2,5)
Equipo sanitario en el que trabaja – n (%)	
Dirección	8 (3,3)
EAP	65 (26,7)
ESF	170 (70,0)
Ocupa un cargo directivo en la AP – n (%)	
Sí	55 (22,6)
No	168 (69,1)
En este momento no, pero lo he ocupado anteriormente	20 (8,2)

^*^DP = desviación estándar.

Los resultados mostraron que la dimensión Motivación para el trabajo presentó los mejores indicadores, con una puntuación total de 47,2 (±8,86) y una media de 3,14 por ítem, lo que refleja una percepción general positiva entre los enfermeros en cuanto a la motivación. Por el contrario, la Seguridad en el Entorno Laboral se clasificó como “atención”, con una puntuación total de 14,6 (±5,66) y una media de 2,43 por ítem, lo que muestra que existen deficiencias en la estructura física y la protección laboral. La gestión estratégica para un trabajo saludable también mostró resultados con puntos de alerta, pero no críticos, con una puntuación total de 36,3 (±12,0) y una media de 2,42 por ítem, lo que indica deficiencias en las políticas de protección de la salud de los trabajadores.

La puntuación general combinada de las tres dimensiones fue de 98,1 (±22,6), con una media de 2,72 por ítem, situándose en el rango “que requiere atención” (73–108) según los parámetros de la EAAT-APS. En general, el entorno evaluado es casi satisfactorio, pero presenta amenazas y elementos que requieren atención e intervención en dos dimensiones. Cada dimensión acompaña este resultado (atención) a pesar de las diferencias identificadas. La Tabla 2 presenta los resultados de las tres dimensiones proporcionadas por la EAAT-APS, con las puntuaciones totales de cada dimensión y la percepción general de los enfermeros sobre su entorno de trabajo.

**Tabla 2:** Resultado de la evaluación del entorno laboral en la atención primaria de salud por ítems y constructos – Manaus, AM, Brasil, 2024.

Ítems	Media ± DE^*^
**Motivación para el trabajo**	Ítems
Lo que hago/practico en mi trabajo le da sentido a mi vida.	3,55 ± 0,72
Me siento involucrado y motivado cuando estoy trabajando.	3,38 ± 0,79
Los compañeros con los que trabajo tienen conocimientos y competencias acordes con el trabajo que realizan.	3,14 ± 0,78
En mi lugar de trabajo, la organización del proceso laboral se lleva a cabo de acuerdo con los objetivos de la Atención Primaria de Salud (APS).	3,12 ± 0,86
Tengo acceso a la información necesaria para realizar mis actividades.	3,41 ± 0,77
Existe una comunicación adecuada y respetuosa entre el equipo y la dirección de la Unidad, lo que fomenta la participación en las decisiones y la organización del trabajo.	3,18 ± 0,96
Mis compañeros actúan de forma integrada (participativa) en el entorno laboral.	2,99 ± 0,88
En mi lugar de trabajo, logramos resolver los conflictos de manera que no perjudiquen los objetivos del trabajo.	3,14 ± 0,80
Existe una comunicación adecuada entre los profesionales de mi lugar de trabajo y los usuarios, lo que favorece el desarrollo del trabajo.	3,06 ± 0,82
Existe coordinación entre la gestión de la Unidad de Salud o las instancias representativas de la gestión de la Atención Primaria y las asociaciones y otras organizaciones colectivas de trabajadores (sindicatos, consejos profesionales, consejo de salud).	2,59 ± 1,14
Cuando trabajo, me siento cómodo y libre de la observación constante por parte de la dirección, la coordinación, los compañeros o los usuarios.	3,15 ± 0,96
Estoy satisfecho con la imagen que los usuarios tienen de mi trabajo.	3,53 ± 0,69
Considero que mis compañeros de trabajo son valorados por los usuarios de la unidad de salud.	2,86 ± 0,90
Considero que las acciones y actitudes de los trabajadores se rigen/protegen por los códigos de ética profesional y las directrices de la Atención Primaria.	3,16 ± 0,83
En mi trabajo convivo con profesionales que presentan ideas o actitudes que favorecen la armonía en el trabajo.	3,01 ± 0,85
Puntuación total de la dimensión (media: 3,14 ± 0,59)	47,2 ± 8,86
**Seguridad en el entorno laboral**
En mi trabajo, puedo organizar mi tiempo y cuidar mis necesidades físicas (por ejemplo, descanso, alimentación e hidratación).	2,60 ± 1,04
La estructura física de mi lugar de trabajo es favorable para el ejercicio profesional.	2,44 ± 1,25
Las condiciones que ofrece mi trabajo me hacen sentir seguro/a y protegido/a.	2,26 ± 1,18
Trabajo en un entorno libre de violencia física contra los trabajadores.	2,52 ± 1,30
Trabajo en un entorno libre de violencia verbal contra los trabajadores.	2,19 ± 1,21
Trabajo en un entorno libre de acoso^*^ contra el trabajador.	2,58 ± 1,22
Puntuación total de la dimensión (media: 2,43 ± 0,94)	14,6 ± 5,66
**Gestión estratégica para un trabajo saludable**
En mi trabajo existen estrategias de atención a la salud del trabajador.	2,02 ± 1,25
En mi trabajo hay apertura para que los trabajadores expresen sus sentimientos, necesidades y valores éticos.	2,58 ± 1,12
Los recursos materiales disponibles en mi lugar de trabajo son suficientes y adecuados para el desempeño de mis actividades.	2,64 ± 0,94
En mi entorno laboral existe una oferta y participación adecuadas de los trabajadores en acciones de formación continua.	2,94 ± 0,94
En mi lugar de trabajo existen protocolos de asistencia que favorecen mis actividades y las de mis compañeros.	3,00 ± 0,97
En mi lugar de trabajo existen intervenciones adecuadas para la protección del trabajador frente a las cargas físicas (por ejemplo: ruido, temperatura, polvo, vibraciones, gases, radiaciones, luminosidad).	2,21 ± 1,20
En mi lugar de trabajo existen intervenciones adecuadas para proteger al trabajador frente a cargas biológicas (bacterias, virus, hongos, parásitos).	2,67 ± 1,17
En mi lugar de trabajo existen medidas adecuadas para proteger a los trabajadores frente a cargas mecánicas (que generan riesgos de fracturas, dolores de columna, miembros, entre otros).	2,10
En mi lugar de trabajo existen medidas adecuadas para proteger a los trabajadores frente a cargas psíquicas (que generan estrés, enfermedades psíquicas, sobrecarga, entre otras).	1,77 ± 1,26
El plan de carrera es conocido y se discute entre los compañeros.	1,94 ± 1,24
Siento que mis derechos como trabajador no corren peligro en mi entorno laboral (por ejemplo: recorte de alguna prestación, suspensión de vacaciones, entre otros).	2,56 ± 1,34
Identifico acciones de prevención de la violencia contra los trabajadores y la promoción de una cultura de paz (por ejemplo, comunicación no violenta) en mi lugar de trabajo.	2,61 ± 1,16
En mi lugar de trabajo identifico políticas y acciones concretas dirigidas a la seguridad y protección de los trabajadores.	2,14 ± 1,24
En mi unidad existen medidas para promover un entorno de trabajo saludable.	2,35 ± 1,20
En mi unidad, los trabajadores pueden tener flexibilidad horaria sin perjuicio salarial.	2,75 ± 1,23
Puntuación total de la dimensión (media: 2,42 ± 0,8)	36,3 ± 12,0
**Puntuación total general (media: 2,72 ± 0,62)**	98,1 ± 22,6

^*^DE = Desviación estándar.

En la Tabla 3, en el análisis de correlación, se observó que la edad (r = –0,203; p = 0,001), el tiempo de actuación en la institución (r_s_ = –0,248; p < 0,001) y el tiempo en la APS (r_s_ = –0,177; p = 0,006) presentaban correlaciones negativas y estadísticamente significativas con la seguridad en el trabajo. Además, el tiempo en la institución se correlacionó negativamente con la gestión estratégica (r_s_ = –0,161; p = 0,012), y el tiempo en la APS mostró una correlación similar (r_s_ = –0,195; p = 0,002). Los ingresos mostraron una correlación negativa débil, estadísticamente significativa, con la motivación para el trabajo (r_s_ = –0,142; p = 0,027), lo que indica que los salarios no se traducen necesariamente en una mayor motivación. En la comparación de medias, el tipo de vínculo laboral temporal se destacó como la única variable categórica con una diferencia significativa, específicamente en la dimensión de gestión estratégica para un trabajo saludable (p = 0,047), lo que muestra que los enfermeros con vínculo laboral temporal evaluaron más positivamente la percepción de la gestión estratégica para un trabajo saludable.

**Tabla 3:** Análisis de correlaciones y asociaciones entre las variables sociodemográficas y las dimensiones de las escalas de evaluación del entorno laboral de los enfermeros en la APS – Manaus, AM, Brasil, 2024.

Variables	Motivación para el trabajo	Seguridad en el entorno laboral	Gestión estratégica para un trabajo saludable	Puntuación total
**Variables continuas**				
Edad (años) – r^*^	0,059 (p = 0,356)	**–0,203 (p = 0,001)**	**–0,132 (p = 0,040)**	–0,098 (p = 0,128)
Tiempo de formación (años) – r_s_^**^	0,061 (p = 0,345)	**–0,162 (p = 0,011)**	–0,122 (p = 0,057)	–0,100 (p = 0,121)
Nivel educativo – r_s_^**^	0,052 (p = 0,421)	–0,037 (p = 0,565)	0,027 (p = 0,678)	0,025 (p = 0,697)
Ingresos – r_s_^**^	**–0,142 (p = 0,027)**	–0,075 (p = 0,243)	–0,082 (p = 0,202)	–0,122 (p = 0,057)
Tiempo de actuación en Atención Primaria (años) – r_s_^**^	0,001 (p = 0,984)	**–0,177 (p = 0,006)**	**–0,195 (p = 0,002)**	**–0,159 (p = 0,013)**
Tiempo de servicio en la institución actual (años) – r_s_^**^	0,059 (p = 0,364)	**–0,248 (p < 0,001)**	**–0,161 (p = 0,012)**	**–0,141 (p = 0,028)**
Carga horaria semanal – r^*^	0,115 (p = 0,074)	–0,112 (p = 0,080)	0,021 (p = 0,747)	0,012 (p = 0,847)
**Variables categóricas**	**Media ± DE**	**Media ± DE**	**Media ± DE**	**Media ± DE**
Sexo				
Masculino	49,0 ± 5,7	15,6 ± 5,1	39,2 ± 11,3	103,7 ± 17,2
Mujer	47,0 ± 9,2	14,4 ± 5,7	35,9 ± 12,1	97,3 ± 23,2
valor p	0,105	0,278	0,154	0,138
Estado civil				
Soltero	45,7 ± 10,1	15,0 ± 5,7	37,1 ± 11,9	97,9 ± 24,2
Casado/Unión estable	47,5 ± 8,1	14,1 ± 5,7	35,4 ± 12,0	97,0 ± 21,9
Divorciado	50,0 ± 8,0	15,6 ± 5,3	38,4 ± 13,0	104 ± 21,4
Viudo	54,0 ± 2,8	18,0 ± 4,2	37,0 ± 2,8	109 ± 4,2
valor p	0,093	0,364	0,579	0,462
Formación				
Licenciatura	47,2 ± 7,3	15,2 ± 6,4	31,8 ± 12,4	94,2 ± 21,2
Residencia	43,3 ± 10,1	14,5 ± 5,3	34,9 ± 9,1	92,7 ± 21,6
Especialización	47,6 ± 8,8	14,7 ± 5,7	36,8 ± 12,1	99,1 ± 22,7
Máster/Doctorado	46,2 ± 8,9	13,2 ± 6,0	33,8 ± 12,7	93,2 ± 23,9
valor p	0,310	0,792	0,462	0,530
Tipo de vínculo				
CLT	45,1 ± 10,5	15,0 ± 6,0	**35,5 ± 13,3^ab^**	95,6 ± 27,2
Estatutario	46,1 ± 7,5	14,4 ± 5,6	**34,4 ± 12,3^a^**	94,9 ± 21,1
Temporal	48,6 ± 9,7	14,7 ± 5,7	**38,3 ± 11,3^b^**	101,6 ± 23,1
valor p	0,073	0,878	**0,047**	0,075
Distrito				
Este	48,7 ± 9,0	14,8 ± 5,4	35,7 ± 11,6	99,1 ± 21,1
Oeste	47,7 ± 8,0	16,4 ± 4,5	38,4 ± 10,7	102,5 ± 19,7
Norte	46,5 ± 8,6	13,6 ± 5,7	35,5 ± 11,6	95,6 ± 21,9
Sur	46,1 ± 9,7	13,8 ± 6,5	36,0 ± 14,1	95,8 ± 27,0
Rural	47,6 ± 10,2	14,8 ± 5,8	35,4 ± 13,3	97,7 ± 24,6
valor p	0,547	0,070	0,708	0,480
Equipo sanitario que trabaja				
Dirección	47,1 ± 10,0	13,6 ± 7,1	37,0 ± 14,7	97,8 ± 28,1
EAP	46,1 ± 7,8	15,6 ± 5,8	36,3 ± 11,6	97,9 ± 20,8
ESF	47,7 ± 9,2	14,3 ± 5,5	36,3 ± 12,1	98,2 ± 23,1
valor p	0,477	0,258	0,986	0,995
Ocupa un cargo directivo en la AP – n (%)				
Sí	48,4 ± 7,7	13,7 ± 5,5	37,6 ± 10,1	99,7 ± 19,1
No	47,2 ± 8,5	14,9 ± 5,6	36,4 ± 12,0	98,4 ± 22,2
En este momento no, pero ya lo he ocupado	44,6 ± 13,7	14,7 ± 6,8	32,2 ± 16,2	91,5 ± 32,7
valor p	0,256	0,445	0,231	0,365

^*^r = coeficiente de Pearson;

^**^r_s_ = coeficiente de Spearman.

## DISCUSIÓN

El predominio de mujeres entre los participantes (87,2 %) es un hallazgo ampliamente reconocido en la literatura sobre el perfil de la enfermería en Brasil y en el mundo. La especialización refleja un alto nivel de cualificación entre los enfermeros de la APS, lo que está en consonancia con las crecientes exigencias en el contexto de la atención sanitaria en la APS. Sin embargo, la baja proporción de profesionales con maestría y doctorado (6,2 % cada uno) puede indicar lagunas en la búsqueda y el acceso a la formación *stricto sensu*, que aún debe fomentarse en las políticas de desarrollo profesional, haciendo frente al desequilibrio regional de la oferta en Brasil. Además, el predominio de los vínculos temporales indica el aumento de este tipo de contratación en diversos niveles y sectores de la salud^(16,17)^.

Los resultados revelaron que, a pesar de un buen nivel de motivación para el trabajo, los enfermeros de la APS de Manaus perciben un entorno de trabajo con fragilidades, especialmente en lo que se refiere a la seguridad en el entorno de trabajo y la gestión estratégica para un trabajo saludable, lo que corrobora estudios internacionales^(18,19)^.

En la dimensión Motivación para el Trabajo, el estudio identificó una puntuación relativamente positiva, pero hay aspectos que sugieren debilidades, con la media más baja en el ítem “integración, participación y armonía en el entorno laboral”, lo que sugiere dificultades en la integración entre compañeros o con la dirección. Esto puede estar relacionado con la sobrecarga de trabajo o la falta de acciones institucionales para fortalecer la unión de los equipos^(20)^.

La motivación de los enfermeros, especialmente en la APS, es un factor que repercute en la productividad y la calidad de la atención. Cuando están motivados, estos profesionales tienden a estar más comprometidos con sus actividades, lo que se traduce en un mejor rendimiento y resultados más positivos para los usuarios. Un hallazgo en Japón, también en una investigación realizada con enfermeros, destacó que este factor actúa como catalizador para el compromiso en el trabajo, ya que los profesionales motivados comprenden la importancia de su papel, se sienten valorados y trabajan en un ambiente colaborativo, además de que tienden a no abandonar la profesión^(20,21)^.

En cuanto al análisis de la dimensión Motivación para el trabajo, en esta muestra de enfermeros que reciben un salario medio superior a la media nacional brasileña, que es de alrededor de tres salarios mínimos y medio^(22)^, la Motivación presentó una correlación negativa con los ingresos, lo que muestra que la motivación no aumenta necesariamente con el aumento del salario. Es importante tener en cuenta que muchos enfermeros sienten que sus salarios no reflejan adecuadamente la carga de trabajo y la responsabilidad que exige el perfil del puesto^(23)^.

La desvalorización salarial da lugar a la percepción de desvalorización de la profesión y puede conducir a la desmotivación, disminuyendo el compromiso con el trabajo. Un estudio cualitativo realizado en 23 municipios del estado de Minas Gerais también mostró la disparidad entre las expectativas salariales y la realidad a la que se enfrentan los profesionales, quienes indicaron que es un factor clave para el descontento y la disminución de la motivación^(24)^. La relación entre ingresos y motivación indica que, aunque la remuneración es un derecho, las políticas de valoración deben ir más allá del salario, invirtiendo en reconocimiento, autonomía y condiciones de trabajo^(6)^.

En la dimensión Seguridad en el Entorno Laboral, la percepción de inseguridad fue evidente, con una puntuación media baja, con puntos de atención para los elementos de estructura física. Los investigadores sostienen que las infraestructuras inadecuadas no solo aumentan el estrés de los profesionales de la salud, sino que también dificultan la práctica de un trabajo seguro y eficaz^(4)^. Los entornos con instalaciones precarias y el déficit o la falta de mantenimiento de las UBS y de los equipos impiden el desempeño satisfactorio de los enfermeros, factor necesario para un entorno de trabajo saludable, lo que aumenta la incidencia de errores y perjudica la atención a los pacientes^(25)^.

Según un estudio realizado en 2024, las mejoras en la infraestructura de las UBS, asociadas a otras intervenciones, contribuyen al bienestar y la calidad de vida de los enfermeros, con un impacto positivo en la salud física y mental en el entorno laboral de la APS^(7)^. Cabe destacar que la infraestructura inadecuada representa una fragilidad en la seguridad del trabajador, un riesgo operativo y un costo para el sistema que debe ser una prioridad de la gestión con una asignación presupuestaria específica^(26)^.

La débil correlación negativa entre la edad y la percepción de la seguridad en el entorno laboral sugiere que los profesionales con más experiencia pueden tener una visión más crítica en la percepción de la exposición a condiciones de trabajo más precarias y a un entorno físico inadecuado, lo que puede exponer a los profesionales a riesgos de accidentes laborales, enfermedades ocupacionales y contaminaciones. Los estudios^(27,28)^ coinciden en este sentido y señalan que los profesionales más experimentados perciben más riesgos y fragilidades en el entorno laboral, lo que puede explicarse por la acumulación de experiencias y una mayor conciencia de los retos a los que se enfrentan en el día a día de la APS. Integrar la percepción del riesgo de los enfermeros experimentados en la planificación estratégica es una forma eficaz de anticipar problemas, mejorar los protocolos de seguridad, aumentar la confianza de todo el equipo y hacer que el entorno de trabajo sea más saludable y seguro para los enfermeros^(27)^.

En este estudio, la exposición a la violencia verbal y física se configura como un resultado significativo en la APS. Los estudios indican que la violencia en el entorno laboral se asocia a menudo con conflictos con los usuarios, sobrecarga de trabajo y falta de medidas de protección^(5)^. La falta de formación adecuada para hacer frente a situaciones de agresión y la ausencia de apoyo institucional agravan la sensación de inseguridad entre los profesionales de la salud, perjudican el bienestar de los enfermeros y también crean un ciclo de insatisfacción^(7,29)^.

Un estudio de enfoque mixto, realizado con enfermeros del contexto hospitalario y de la APS, demuestra que la implementación de protocolos de comunicación colaborativa y la creación de redes de apoyo entre pares pueden ser estrategias eficaces para reducir la violencia contra los enfermeros en la APS, más allá de la capacitación individual y el apoyo institucional tradicional. La investigación evidencia que el fortalecimiento de las relaciones interprofesionales y la construcción de un ambiente de trabajo basado en la solidaridad y el apoyo mutuo entre colegas contribuyen significativamente a reducir la vulnerabilidad de los profesionales y a mejorar la salud mental de los enfermeros^(5)^.

Los hallazgos en la dimensión Gestión Estratégica para el Trabajo Saludable evidencian lagunas significativas en las prácticas institucionales de cuidado del trabajador. Uno de los puntos críticos es la insuficiencia de intervenciones contra las cargas psíquicas. Esta relación se encuentra en el contexto de la sobrecarga de trabajo y el estrés, situaciones frecuentes en la APS, principalmente en enfermeros, con un impacto negativo en la salud mental y el rendimiento. La falta de estrategias adecuadas para abordar estas cuestiones crea un ciclo adverso y da lugar a profesionales agotados, con trastornos del sueño y disfunciones sociales^(30,31)^. Deben implementarse algunas medidas, como cambios en las políticas laborales, reducción de la carga de trabajo y/o horarios flexibles, mejoras en la relación entre compañeros y el equipo directivo y el establecimiento de centros/puntos de asesoramiento mental intrainstitucionales. Se trata de estrategias que tienen por objeto crear un entorno de trabajo más saludable, fortaleciendo la resiliencia de los enfermeros y atenuando los efectos negativos de las cargas psíquicas^(31)^.

La dimensión Gestión Estratégica para el Trabajo Saludable se mostró asociada al tipo de vínculo laboral. Los trabajadores con vínculos presentan mejores percepciones sobre la Gestión Estratégica, con especial destaque para los trabajadores temporales. Esta asociación puede reflejar diferencias en las expectativas entre los profesionales temporales y los estatutarios, así como un posible temor a evaluar críticamente la gestión, a pesar de la garantía de anonimato en las respuestas, por estar en vínculos con plazo determinado; así, sus evaluaciones pueden verse influenciadas por la necesidad de preservar las relaciones a largo plazo dentro de la institución^(32)^.

Las contrataciones temporales, aunque a veces son necesarias para cubrir vacantes en regiones con escasez de enfermeros, comprometen la estabilidad a largo plazo y la retención de profesionales. Este escenario exige políticas que prioricen los vínculos estables, especialmente en áreas específicas de Brasil, para garantizar una fuerza laboral calificada y motivada. La literatura sugiere invertir en concursos públicos y en la fijación de profesionales, promoviendo una mayor calidad en la continuidad de la atención^(16,17)^.

La puntuación general de la EAAT-APS mostró una evaluación global regular del entorno de trabajo. Aunque los enfermeros muestran motivación por el trabajo, las dimensiones relacionadas con la seguridad y la gestión estratégica indican áreas que requieren atención y mejoras para garantizar un entorno de trabajo más saludable y seguro. Estas dimensiones objetivas y subjetivas son fundamentales para garantizar la calidad de las prácticas profesionales de los enfermeros en la APS, con interfaces en un clima de trabajo positivo capaz de promover la salud de los enfermeros y un clima institucional que favorezca la calidad de la atención^(10,11,12)^. Cabe señalar que las prácticas y condiciones de trabajo de los profesionales de enfermería pueden variar ampliamente entre las diferentes unidades de APS debido a las variaciones en las políticas locales, la infraestructura y la gestión^(33)^.

Una limitación a considerar es que, aunque se realizó la estratificación por distritos, no se aplicó la aleatorización dentro de cada estrato. Este aspecto puede haber influido en los resultados, ya que la elección de los participantes puede no haber reflejado de manera equilibrada la diversidad presente en cada distrito, lo que puede afectar a la generalización de los hallazgos. Además, más de la mitad de los profesionales participantes fueron contratados con régimen temporal, lo que puede influir en los resultados en uno de los ámbitos evaluados por el instrumento (gestión estratégica para un trabajo saludable), teniendo en cuenta las especificidades y posibles inestabilidades asociadas a este tipo de vínculo laboral.

## CONCLUSIÓN

Los hallazgos de esta investigación evidenciaron una percepción general regular/moderada del entorno laboral de los enfermeros de la APS de Manaus, Brasil. Aunque presentan un buen nivel de motivación en el trabajo, aspectos como la seguridad en el entorno laboral y la gestión estratégica para un trabajo saludable fueron evaluados negativamente. Además, los análisis inferenciales demostraron asociaciones significativas entre las características sociodemográficas y las dimensiones de la escala EAAT-APS, lo que indica que los factores individuales y organizacionales pueden influir en la percepción y los significados atribuidos al entorno laboral. Los resultados destacan la necesidad de intervenciones centradas en mejorar la seguridad y la gestión estratégica, con el fin de promover un entorno más favorable para la práctica profesional en la APS.

Se evidencian desafíos, como la falta de integración entre colegas y la comunicación organizacional, la percepción de inseguridad en el entorno laboral, reflejada en la evaluación de la estructura física y la protección contra la violencia, con una necesidad urgente de mejoras en los entornos laborales. Además, la gestión estratégica para un trabajo saludable sigue siendo deficiente, con la ausencia de algunas iniciativas institucionales destinadas a mejorar el entorno laboral en la APS. Estrategias como las inversiones en infraestructura, el fortalecimiento de la gestión participativa y la ampliación de las acciones de protección de la salud de los trabajadores pueden contribuir a minimizar los impactos negativos. Además, es fundamental promover la valoración profesional, con incentivos a la cualificación, salarios adecuados y estabilidad en el empleo.

## Data Availability

Todo el conjunto de datos que apova los resultados de este estudio fue publicado en el propio artículo.
